# Shear Stress Drives the Cleavage Activation of Protease‐Activated Receptor 2 by PRSS3/Mesotrypsin to Promote Invasion and Metastasis of Circulating Lung Cancer Cells

**DOI:** 10.1002/advs.202301059

**Published:** 2023-07-03

**Authors:** Muya Zhou, Koukou Li, Kathy Qian Luo

**Affiliations:** ^1^ Department of Biomedical Sciences, Faculty of Health Sciences University of Macau Taipa Macao SAR 999078 China; ^2^ Ministry of Education Frontiers Science Center for Precision Oncology University of Macau Taipa Macao SAR 999078 China

**Keywords:** cancer metastasis, circulating tumor cells, protease‐activated receptor 2, PRSS3/mesotrypsin, shear stress

## Abstract

When circulating tumor cells (CTCs) travel in circulation, they can be killed by detachment‐induced anoikis and fluidic shear stress (SS)‐mediated apoptosis. Circulatory treatment, which can make CTCs detached but also generate SS, can increase metastasis of cancer cells. To identify SS‐specific mechanosensors without detachment impacts, a microfluidic circulatory system is used to generate arteriosus SS and compare transcriptome profiles of circulating lung cancer cells with suspended cells. Half of the cancer cells can survive SS damage and show higher invasion ability. Mesotrypsin (PRSS3), protease‐activated receptor 2 (PAR2), and the subunit of activating protein 1, Fos‐related antigen 1 (FOSL1), are upregulated by SS, and their high expression is responsible for promoting invasion and metastasis. SS triggers PRSS3 to cleave the N‐terminal inhibitory domain of PAR2 within 2 h. As a G protein‐coupled receptor, PAR2 further activates the G*α*_i_ protein to turn on the Src‐ERK/p38/JNK‐FRA1/cJUN axis to promote the expression of epithelial–mesenchymal transition markers, and also PRSS3, which facilitates metastasis. Enriched PRSS3, PAR2, and FOSL1 in human tumor samples and their correlations with worse outcomes reveal their clinical significance. PAR2 may serve as an SS‐specific mechanosensor cleavable by PRSS3 in circulation, which provides new insights for targeting metastasis‐initiating CTCs.

## Graphical abstract

Circulating tumor cells (CTCs) encounter detachment and shear stress (SS) in bloodstream. A microfluidic circulatory system is developed and circulated cancer cells display higher metastatic abilities than detached cells. SS triggers PRSS3 to cleave and activate protease‐activated receptor 2 (PAR2), a G protein‐coupled receptor which activates G*α*_i_‐Src‐ERK/p38/JNK‐FRA1/cJUN axis to facilitate metastasis. PAR2 may be a SS‐specific mechanosensor in metastasis‐initiating CTCs.

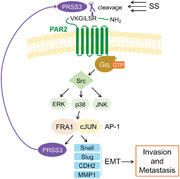

## 1 Introduction

Cancer has long been a life‐threatening disease. Among cancer‐related deaths, most occur due to metastasis, and circulating tumor cells (CTCs) are the major source.^[^ ^1^ ^]^ Evidence has shown that higher CTC numbers in blood correlate with worse clinical outcomes in patients with breast cancer, lung cancer, colorectal cancer, prostate cancer and others.^[^ ^2^, ^3^, ^4^, ^5^ ^]^ Several studies observed metastatic tumors after injecting CTCs isolated from patient blood or mouse blood into mice, demonstrating the importance of CTCs in metastasis.^[^ ^4^, ^6^, ^7^, ^8^, ^9^ ^]^

When CTCs enter blood circulation, they encounter three major challenges. Anoikis mainly accounts for the death of CTCs, which refers to a form of programmed cell death due to detachment from the previous living environment. Attack from the immune system and fluid shear stress (SS) in bloodstream can cause cell death in CTCs as well.^[^ ^10^ ^]^ As a result, most CTCs die in circulation, and only a few can successfully extravasate blood vessels and form colonies in secondary organs.

During circulation, the mechanical stress generated from blood flow not only kills CTCs, but also promotes metastasis.^[^ ^11^, ^12^, ^13^, ^14^ ^]^ Therefore, understanding the underlying mechanisms is important for developing new therapies to prevent metastasis and benefit patients. To investigate the effects of SS on CTCs, researchers have developed various in vitro models to mimic blood SS, including stirring cells in suspension,^[^ ^15^ ^]^ rotating cells,^[^ ^16^ ^]^ cone and plate viscometers,^[^ ^17^, ^18^ ^]^ syringes and needles,^[^ ^19^, ^20^ ^]^ parallel plate flow chambers,^[^ ^21^ ^]^ and microfluidic devices.^[^ ^22^, ^23^ ^]^ When stirring or rotating cells, the levels of SS are determined by revolutions per minute. While in most of the models that use tubes to mimic circulation, the levels of SS are calculated based on flow types (laminar or turbulent), viscosity of the fluid, flow rates, and diameters of the vessels. Under physiological conditions, the levels of SS can range from 1 to 4 dyne cm^−2^ in veins, 4 to 30 dyne cm^−2^ in arteries, 10 to 20 dyne cm^−2^ in capillaries and can reach up to 60 dyne cm^−2^ when the person is doing exercise.^[^ ^13^, ^24^ ^]^

With the help of in vitro models, it has been reported that SS induces cell death in different kinds of cancers, and the sensitivity to SS damage varies among cell types. Usually, cells with higher malignancy can survive better.^[^ ^23^, ^25^ ^]^ Several oncogenes, including caveolin‐1, focal adhesion kinase (FAK), Rho‐associated coiled‐coil containing protein kinase 1, Ras, Myc, and phosphoinositide 3‐kinase (PI3K), have displayed the ability to increase cell survival under SS.^[^ ^16^, ^19^ ^]^ Activation of the atonal bHLH transcription factor 8 or the RhoA‐myosin II axis and the nuclear localization of lamin A/C also contribute to the SS resistance of CTCs.^[^ ^20^, ^26^, ^27^, ^28^ ^]^ In addition, elevation of ß‐globin or manganese superoxide dismutase help CTCs withstand the damage of SS‐induced reactive oxygen species.^[^ ^22^, ^29^ ^]^

Once CTCs survive in circulation, they will migrate and invade through endothelial cells and colonize at distant organs to develop metastasis. SS promotes the epithelial–mesenchymal transition (EMT), migration and invasion in esophageal cancer, breast cancer, lung cancer, prostate cancer and liver cancer cells.^[^ ^11^, ^30^, ^31^, ^32^, ^33^ ^]^ By injecting tumor cells into zebrafish, extravasation of CTCs was observed clearly in vivo.^[^ ^22^ ^]^ Yes‐associated protein 1 was identified as a fluid mechanosensor and displayed an executive role in invasion, modulated by the Rho kinase‐cofilin signaling axis.^[^ ^31^ ^]^ Activation of Ras, Src, FAK, extracellular signal‐regulated kinase (ERK), and manganese superoxide dismutase favored this process as well.^[^ ^11^, ^21^, ^30^, ^34^ ^]^ In addition to the improved cell migration and invasion abilities, cancer cells also showed increased stemness and chemoresistance under SS.^[^ ^22^, ^35^, ^36^ ^]^

Previously, our group developed a microfluidic circulatory system to mimic SS in vitro and found that during circulation, CTCs managed to associate with each other and form clusters to struggle with SS destruction and gain metastatic ability, which was facilitated by high expression of desmosomal proteins desmocollin‐2 and plakophilin‐1.^[^ ^14^ ^]^ Previous studies have mainly compared circulating cancer cells with adherent cells or applied shear flow over adherent cells, while in circulation, cells are challenged with both detachment and SS. In addition, it has been reported that the suspension state was also able to enhance the cell migration, invasion, and lung metastatic abilities of breast cancer cells.^[^ ^37^ ^]^ Therefore, it is necessary to exclude the impacts of suspension to explore the distinct effects of SS on cancer cells. To date, how cancer cells perform differently in suspension and SS conditions and the underlying mechanisms of SS‐promoted metastasis have not been well studied. Here, we made comparisons among untreated, suspended and circulating cancer cells to figure out the SS‐specific effects on cancer cells and elucidated the potential mechanisms that would help to target metastasis‐initiating CTCs.

## 2 Results

### 2.1 SS Has Greater Effects than Detachment in Inducing Cell Death and Enhancing Colony Formation, Migration, and Invasion

Previously, we developed a microfluidic circulatory system that uses a peristaltic pump to generate a pulsatile flow, mimicking the blood SS that cancer cells encounter during circulation (Figure S1, Supporting Information).^[^ ^11^, ^14^, ^22^ ^]^ However, in circulation, not only SS but also detachment could influence cancer cells. To rule out the impacts of detachment on CTCs, we seeded cells in an ultralow attachment 6‐well plate coated with a hydrogel layer to achieve suspension conditions as a control (Figure S1, Supporting Information). In accordance with our previous study, cancer cells were treated with SS at the level of 15 dyne cm^−2^ (SS15), which represents the average SS level in human arteries.^[^ ^14^, ^19^ ^]^

**Figure 1:**
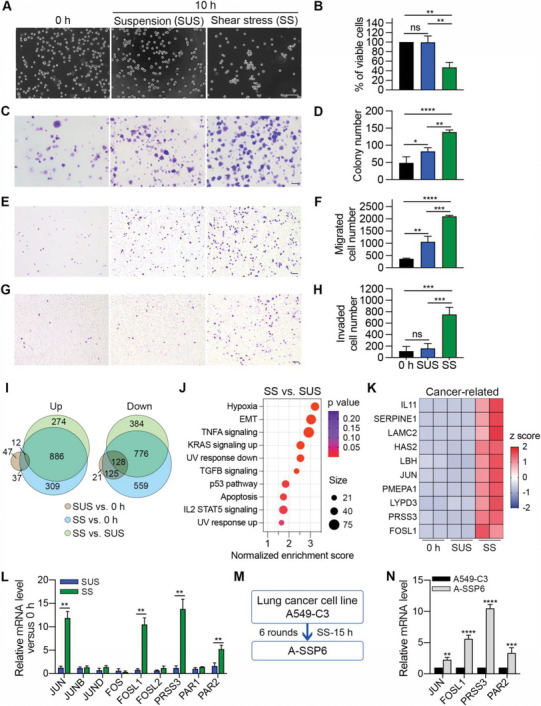
SS induced cell death and elevated cell colony formation, migration and invasion abilities compared with both 0 h and suspension conditions. A) Phase images of A549 cells at 0 h and after 10 h of suspension and SS treatment. Scale bar: 100 µm. B) The MTT assay was used to evaluate the viability of A549 cells under the indicated conditions. C,D) Representative images and quantification results of the colony formation assay for A549 cells under the indicated conditions. 1000 cells were seeded in each well of 6‐well plates and allowed to grow for 10 days. Scale bar: 2 mm. E,F) Representative images and quantification results of the Transwell migration assay for A549 cells under the indicated conditions. 10 000 cells were seeded and allowed to migrate for 14 h. Scale bar: 100 µm. G,H) Representative images and quantification results of the Transwell invasion assay for A549 cells under the indicated conditions. 20 000 cells were seeded and allowed to invade for 14 h. Scale bar: 100 µm. I) Venn diagrams of differentially expressed genes in the indicated comparisons. The thresholds were set as *p* < 0.05, a fold change of ≥2 for upregulated genes and a fold change of ≤0.5 for downregulated genes. J) Gene Set Enrichment Analysis (GSEA) of differentially expressed genes in the SS group compared with the suspension group (SUS). Gene sets with the top 10 highest normalized enrichment scores are shown. K) Expression heatmap of the 10 most upregulated genes in RNA‐seq that have been reported to promote cancer progression. L) qPCR results showing the relative mRNA levels of JUN, JUNB, JUND, FOS, FOSL1, FOSL2, PRSS3, PAR1, and PAR2 in A549 cells under suspension and SS conditions in comparison with those at 0 h. M) Schematics of generating the SS‐resistant cell line A‐SSP6 from the parental lung cancer cell line A549‐C3 through six rounds of circulation. N) qPCR results showing the relative mRNA levels of JUN, FOSL1, PRSS3, and PAR2 in A‐SSP6 cells compared to A549‐C3 cells. The quantification results are the means ± SD from three independent experiments. Significant differences were determined by one‐way ANOVA (B,D,F,H) and two‐way ANOVA (L,N). *p < 0.05, **p < 0.01, ***p < 0.001, and ****p < 0.0001. ns, not significant.

After suspension or SS treatment, non‐small cell lung cancer (NSCLC) A549 cells were collected and pipetted for imaging, and cell viability was measured by the 3‐(4,5‐dimethylthiazol‐2‐yl)‐2,5‐diphenyltetrazolium bromide (MTT) assay. From the phase images, we observed a significant reduction in existing cells and the formation of tight clusters under SS compared with 0 h and suspension conditions (**Figure** **1**A). The quantified results showed that SS killed 53% of the circulating cancer cells, while cell viability did not change significantly in the suspension state (Figure 1B). Moreover, the colonies formed by A549 cells increased 1.7‐fold after they were suspended for 10 h, and SS further increased the colony formation ability of A549 cells to another 1.7‐fold compared with the suspension condition (Figure 1C,D).

To further explore how SS affects cancer cells, we used Transwell assays to detect the migration and invasion abilities of A549 cells. As a result, suspension treatment enhanced cell migration to 2.9‐fold compared with normally cultured cells, while SS further doubled the migrated cell number (Figure 1E,F). In terms of invasion, SS markedly elevated the invaded cell number to 4.7‐fold compared with suspension conditions, but no significant changes were observed in cells before and after suspension treatment (Figure 1G,H).

To confirm that the SS effects we observed are not cell line specific, we suspended and circulated NSCLC H1975 cells, triple‐negative breast cancer MDA‐MB‐231 cells and MDA‐MB‐468 cells and colorectal cancer HCT‐116 cells for 10 h and measured the cell viability using the MTT assay first. The results showed that similar to what happened in A549 cells, SS killed 43% to 67% of these cancer cells, while leaving cells in suspension barely impaired cell viability (Figure S2A, Supporting Information). We also observed that SS could significantly increase the colony formation abilities of these cancer cells to more than 2‐fold compared with suspension conditions (Figure S2B, Supporting Information). In addition, compared with suspension conditions, SS enhanced cell migration to 1.5‐ to 2.2‐fold in H1975, MDA‐MB‐231, MDA‐MB‐468, and HCT116 cells, while the invasion ability of these four cell lines had a greater elevation of 2.2‐ to 2.6‐fold after SS treatment (Figure S2C,D, Supporting Information). Together, we can conclude that excluding the impacts of detachment, SS could particularly kill circulating cancer cells, while cancer cells that successfully survived SS damage gained intensified colony formation, migration and invasion abilities.

### 2.2 Identification of the Key Genes That Are Differentially Expressed during Circulation

To investigate how SS promoted the metastatic potential of cancer cells and identify critical genes during this process, we performed RNA‐seq analysis to compare the gene expression profiles of A549 cells under 0 h, suspension and SS conditions. Genes with a fold change ≥2 or ≤0.5 and *p* < 0.05 were considered differentially expressed genes. Following these criteria, we found that suspension conditions induced the upregulation of 96 genes and downregulation of 274 genes compared with those at 0 h. In the SS group, 1224 genes were increased, and 1588 genes were decreased transcriptionally compared with those at 0 h. Importantly, in comparison with suspension conditions, 1172 genes were upregulated, and 1288 genes were downregulated after SS treatment (Figure S3, Supporting Information).

To determine the distinct effects of SS on gene expression, we focused on the genes that were differentially expressed under SS compared with both 0 h and suspension conditions but were not significantly changed before and after suspension treatment. Venn diagrams were drawn, and 886 upregulated and 776 downregulated genes were identified (Figure 1I). Through gene set enrichment analysis, we found that many of the 1662 differentially expressed genes belonged to the EMT gene set, which was supposed to play a crucial role in cancer metastasis. Other cancer progression‐related pathways were enriched as well, such as the Kirsten rat sarcoma virus and transforming growth factor beta signaling pathways (Figure 1J). Together, these results showed that SS uniquely led to dramatic changes in gene expression in circulating cancer cells. In addition, many of the differentially expressed genes are related to cancer progression and metastasis, which is consistent with the SS‐elevated cell migration, invasion, and colony formation abilities that we observed above.

Next, to identify several potential genes for further investigation, we selected the top ten genes from the 886 upregulated genes with fragments per kilobase of exon per million mapped fragments values greater than 1, which suggests their detectability (Figure 1K). Interestingly, we found that two subunits of the transcription factor activator protein 1 (AP‐1), jun proto‐oncogene (JUN) and FOS‐like 1 (FOSL1), appeared in this list, indicating the importance of AP‐1 during SS. We also obtained another attractive gene, serine protease 3 (PRSS3), which has not been well studied in cancer but was highly upregulated by SS (Figure 1K). Following PRSS3, we searched the RNA‐seq data and found that the potential targets of PRSS3, protease‐activated receptor 1 and 2 (PAR1 and PAR2), were also significantly increased under SS compared with suspension conditions. Then, we used quantitative polymerase chain reaction (qPCR) to detect the mRNA levels of subunits of AP‐1, including JUN, JUNB, and JUND from the Jun family and FOS, FOSL1, and FOSL2 from the Fos family, as well as PRSS3, PAR1, and PAR2. Compared with suspension conditions, SS significantly elevated the mRNA levels of JUN, FOSL1, PRSS3, and PAR2 to 11.9‐, 10.5‐, 13.8‐, and 5.2‐fold, respectively (Figure 1L).

Previously, we generated an SS‐resistant A‐SSP6 cell line by circulating parental A549‐C3 cells under 15 h of SS treatment and then allowed them to grow for six rounds, which is also more metastatic than its parental cell line.^[^ ^14^ ^]^ To determine whether the expression level changes of these genes are only transient effects or can be stabilized, we examined the mRNA levels of JUN, FOSL1, PRSS3, and PAR2 in A549‐C3 and A‐SSP6 cells. The results demonstrated that these four genes were stably elevated in A‐SSP6 cells, among which PRSS3 had the highest 10.5‐fold increase, followed by FOSL1 with a 5.6‐fold increase (Figure 1N). To summarize, PRSS3, PAR2, FOSL1, and JUN were selected from our RNA‐seq data for their high fold changes after SS and in SS‐resistant metastatic cells.

### 2.3 PRSS3, PAR2, FOSL1, and JUN Are Elevated and Activated in Response to SS

**Figure 2:**
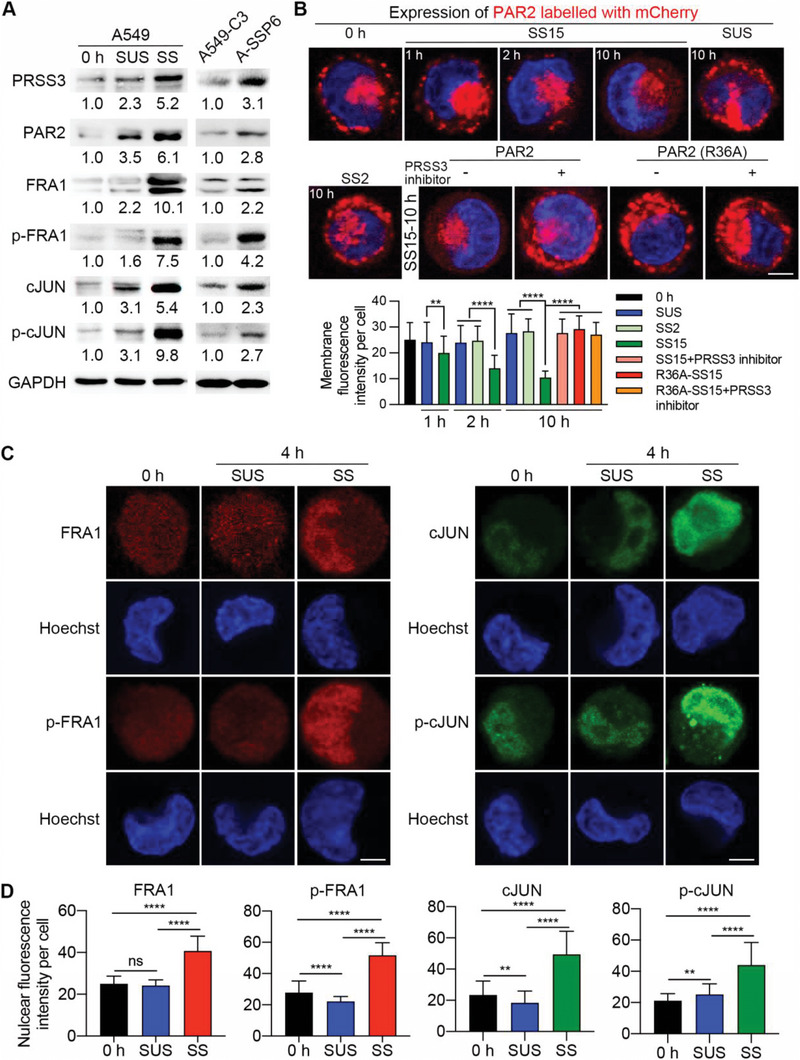
Upregulation of PRSS3, PAR2, FOSL1, and JUN induced by SS was further verified. A) Western blots showing the protein levels of PRSS3, PAR2, FRA1, p‐FRA1, cJUN, and p‐cJUN in A549 cells under the indicated conditions (left) and in A549‐C3 and A‐SSP6 cells (right). B) The cleavage of N‐mCherry‐PAR2 in A549 cells was observed under a confocal microscope at 0 h and after 1, 2, or 10 h of treatment (upper). Red fluorescence appeared on the cell membrane when PAR2 was not cleaved, while its significant reduction indicated cleavage. “PAR2 (R36A)” refers to A549 cells expressing N‐mCherry‐PAR2 with an R36A mutation to prevent cleavage under SS, while A549 cells expressing wild‐type PAR2 were used under other conditions. Cells were pretreated and cocirculated with or without 10 µm PRSS3 inhibitor diminazene. Scale bar: 5 µm. The red fluorescence intensity of the membrane under each condition was measured (lower; *n* ≥ 50 cells). C) Representative immunofluorescence (IF) staining images showing the subcellular localization of FRA1, p‐FRA1, cJUN, and p‐cJUN before and after 4 h of suspension and SS treatment. Scale bar: 5 µm. D) Quantified nuclear fluorescence intensity for A549 cells in (C) (*n* ≥ 100 cells). The quantification results are the means ± SD from three independent experiments. Significant differences were determined by one‐way ANOVA (B,D). **p < 0.01 and ****p < 0.0001. ns, not significant.

In addition to the mRNA level changes, we also detected the protein levels of PRSS3, PAR2, FOSL1, and JUN through western blotting. Since the subunits of AP‐1 need to be phosphorylated to function as a transcription factor, we also examined the levels of phosphorylated forms of FOSL1‐ and JUN‐encoded proteins. Compared with those at 0 h, the levels of PRSS3, PAR2, Fos‐related antigen 1 (FRA1, encoded by FOSL1), phosphorylated FRA1 (p‐FRA1), cJUN (encoded by JUN), and phosphorylated cJUN (p‐cJUN) were elevated 2.3‐ to 3.5‐fold in the suspension state and 5.2‐ to 10.1‐fold by SS. Particularly, the results showed that SS increased the levels of both FRA1 and its phosphorylated form to 4.6‐ to 4.7‐fold in comparison with suspension conditions, while cJUN and phosphorylated cJUN only increased to 1.7‐ and 3.2‐fold, respectively (**Figure** **2**A). This suggests that FOSL1 had a stronger response to SS than JUN. In more metastatic A‐SSP6 cells, these six proteins were 2.2‐ to 4.2‐fold higher than those in parental A549‐C3 cells. The upregulation of the protein levels of FOSL1 and JUN was similar, but phosphorylated FRA1 had a higher increase (4.2‐fold) than phosphorylated cJUN (2.7‐fold) (Figure 2A).

Higher protein levels of PRSS3, PAR2, FRA1, p‐FRA1, cJUN, and p‐cJUN were also detected in cells of another lung cancer cell line H1975 after circulation (Figure S4A, Supporting Information). In addition, we searched for the levels of PRSS3, PAR2, FOSL1, and JUN in the RNA‐seq data of the breast cancer cell lines MCF7‐C3 and MDA‐MB‐231‐C3 (231‐C3).^[^ ^14^, ^38^ ^]^ One previous study from our group has revealed the higher metastatic ability of 231‐C3 cells compared with MCF7‐C3 cells.^[^ ^22^ ^]^ All four genes had higher mRNA levels in 231‐C3 cells, suggesting the positive correlation of these four genes with cancer metastasis (Figure S4B, Supporting Information). Together, our results indicate that the upregulation of PRSS3, PAR2, FOSL1, and JUN and the activation of AP‐1 were verified at both the mRNA and protein levels.

We then used immunofluorescence staining to observe the distribution of the upregulated proteins in A549 cells before and after suspension or SS treatment. PAR2 is a transmembrane protein, and the immunofluorescence results showed a significant increase in fluorescence intensity outside the nucleus under SS compared with 0 h and suspension conditions (Figure S5A, Supporting Information). Moreover, PAR2 is a G protein‐coupled receptor (GPCR) activated through the proteolytic cleavage of its N‐terminal region of 36 amino acids by proteases, including the member of the trypsin family, PRSS3, to motivate downstream signaling pathways. To test whether cleavage occurred during circulation, we designed a construct in which we inserted the red fluorescent protein mCherry into the N‐terminus of PAR2 upstream of the trypsin cleavage site (N‐mCherry‐PAR2) as previously described to visualize cleavage (Figure S5B, Supporting Information).^[^ ^39^, ^40^ ^]^ Members of the trypsin family have been reported to cleave PAR2 after the Arg36 residue.^[^ ^41^, ^42^ ^]^ To better validate the cleavage, we also designed a construct expressing N‐mCherry‐PAR2 with an R36A mutation, which could disable cleavage by trypsin.

A549 cells were transfected with the two constructs and subjected to suspension and circulatory treatment. As a result, in A549 cells expressing wild‐type PAR2, red signals existed on the cell membrane under 0 h and suspension conditions. The membrane fluorescence intensity slightly declined after 1 h of circulation, while it significantly decreased by 40% at 2 h. Ten hours of SS treatment further reduced the red fluorescence on the cell membrane by 62% (Figure 2B). To further explore if PAR2 can serve as a mechanosensor responding to SS, we circulated the cells under a low level of SS at 2 dyne cm^−2^ (SS2), which represents the average SS level in human veins. The results showed the membrane fluorescence was not reduced under SS2 treatments (2 h, 10 h) compared with 0 h and suspension conditions (2 h, 10 h), indicating that different from SS15, a veinous level of SS (SS2) was not able to trigger the cleavage of PAR2 (Figure 2B). These results demonstrated that arteriosus SS could effectively induce the cleavage of PAR2 as early as 2 h, while cells treated without SS or with low SS could not, suggesting the potential of PAR2 to be a mechanosensor in response to arteriosus SS. Besides, we also detected the protein levels of PRSS3 under SS2 and SS15, and found that when cells were treated with SS15, the level of PRSS3 was elevated compared with 0 h, suspension and SS2 conditions (Figure S5C, Supporting Information). These results were consistent with the observation that PAR2 could be cleaved in 2 h under SS15 and this fast cleavage may be due to the higher stability of PRSS3 under higher levels of SS.

To verify the cleavage of PAR2 by PRSS3, we used the PRSS3 inhibitor diminazene to treat A549 cells expressing N‐mCherry‐PAR2 under SS. The fluorescent images and quantified membrane fluorescence intensity showed that when PRSS3 was inhibited, the red fluorescence on the cell membrane significantly increased to 2.8‐fold, indicating the suppression of cleavage. In contrast, in cells expressing N‐mCherry‐PAR2 with the cleavage‐blocking R36A mutation, red signals existed on the cell membrane regardless of whether the cells were treated with diminazene (Figure 2B). Hence, we can conclude that PAR2 is cleaved by PRSS3 after the Arg36 residue early during circulation, and this is an SS‐specific cleavage that does not occur in the suspension state.

As subunits of the transcription factor AP‐1, activated FRA1 and cJUN need to enter the nucleus and regulate the transcription of target genes. Nuclear translocation of FRA1 and p‐FRA1 was obviously observed under SS, and the nuclear fluorescence intensity was enhanced remarkably as well. cJUN and p‐cJUN were mainly distributed in the nucleus under all conditions, while the nuclear fluorescence intensity was elevated by SS but not in the suspension state (Figure S5D, Supporting Information). These results showed that high levels of FRA1 and cJUN, as well as their activated forms, localize in the nuclei of cells after 10 h of SS treatment compared with suspension conditions. We further found that SS caused the nuclear translocation of FRA1 and p‐FRA1 and higher expression levels of cJUN and p‐cJUN in the nucleus as early as 4 h (Figure 2C). Consistently, the quantified results showed that the nuclear fluorescence intensities of FRA1, p‐FRA1, cJUN, and p‐cJUN were elevated approximately twofold by SS at 4 h (Figure 2D).

Since FRA1 and cJUN dimerize to form AP‐1 and FRA1 had a higher elevation (4.6‐fold) than cJUN (1.7‐fold) between SS and suspension conditions in A549 cells (Figure 2A), we focused on the role of FRA1‐coding gene (FOSL1) in the following study. Next, we suspended and circulated A549 cells for 3, 5, 8, 10, and 12 h to determine the changes in these key molecules over time. We examined the relative mRNA levels of PRSS3, PAR2, and FOSL1, and the results showed that 5 h of SS was already able to induce the elevation of FOSL1, while PRSS3 and PAR2 started to have a significant upregulation after 8 h of circulation (Figure S5E, Supporting Information). Collectively, the higher expression levels of PRSS3, PAR2, FOSL1, and JUN, cleavage of PAR2 by PRSS3, and activation of FOSL1 and JUN after circulation were well validated.

### 2.4 Knockdown of PRSS3, PAR2, and FOSL1 Impairs the Invasion and Metastatic Abilities of SS‐Resistant A‐SSP6 Cells

To determine whether the three selected genes were responsible for SS‐triggered cell migration and invasion, we designed short hairpin‐mediated RNAs (shRNAs) to lower their expression levels in SS‐resistant A‐SSP6 cells. Two shRNAs were designed for PRSS3 and FOSL1 separately, while only one shRNA was designed for PAR2 because it was supposed to be the target of PRSS3. The knockdown efficiencies were assessed using qPCR and western blotting. The results showed that at least one shRNA could effectively reduce both the mRNA and protein levels for each gene (Figure S6A, Supporting Information).

After they were transfected with shRNAs, A‐SSP6 cells were first circulated for 10 h to test the SS survival rate after knockdown. As a result, in A‐SSP6 cells with lower expression levels of PRSS3, PAR2, and FOSL1, only ≈30% of cells survived under SS, while nearly 70% of the A‐SSP6 cells transfected with the control shRNA survived (Figure S6B, Supporting Information). In addition, we assessed the colony formation ability of these A‐SSP6 cells and found that after knocking down PRSS3 and FOSL1, the colony numbers were reduced by nearly 50%, while after knocking down PAR2, it was only reduced by 30% compared with the control group (Figure S6C, Supporting Information).

**Figure 3:**
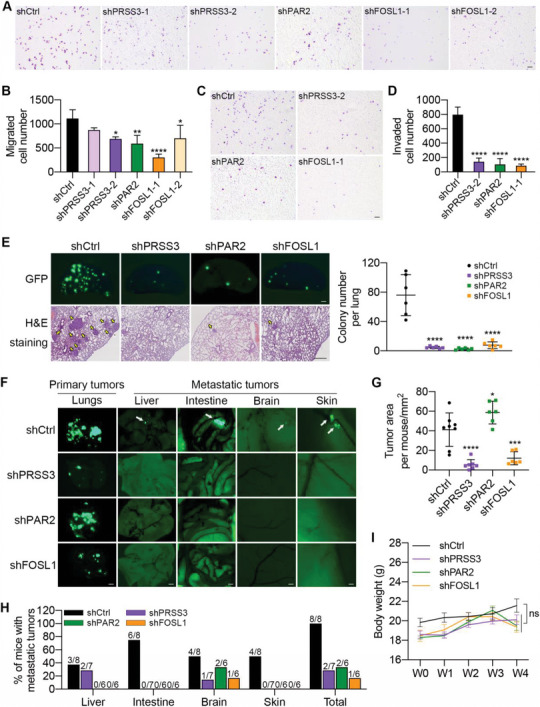
Knockdown of PRSS3, PAR2, and FOSL1 impaired the metastatic ability of A‐SSP6 cells. A,B) Representative images and quantification results of migrated A‐SSP6 cells after knocking down PRSS3, PAR2, and FOSL1 using shRNAs. 10 000 cells were seeded and allowed to migrate for 18 h. Scale bar: 100 µm. C,D) Representative images and quantification results of the invaded A‐SSP6 cells transfected with shRNAs. 20 000 cells were seeded in each insert and allowed to invade for 18 h. Scale bar: 100 µm. E) Representative fluorescent and H&E staining images and quantification results of colonies per left lung 28 days post injection. One million cells were injected into the tail vein of each NOD/SCID mouse (*n* = 6 mice per group). Scale bar: 1 mm for GFP images and 400 µm for H&E staining images. F) Representative fluorescent images of primary tumors in the lungs and metastatic tumors in the liver, intestine, brain and skin (*n* = 6–8 mice per group). Four million cancer cells were orthotopically injected into the right lungs of each NOD/SCID mouse. Scale bar: 2 mm (lungs, liver, and intestine) and 200 µm (brain and skin). White arrowheads indicate metastatic tumors. G) The tumor area in all five lungs of each mouse was measured by ImageJ. H) The quantified percentage of mice with metastatic tumors in each group 4 weeks after orthotopic injection was calculated. I) The body weights of each mouse at 0, 1, 2, 3, and 4 weeks after orthotopic implantation were measured. The quantification results are the means ± SD from three independent experiments or from more than five mice. Significant differences were determined by one‐way ANOVA (B,D,E,G) and two‐way ANOVA (I). *p < 0.05, **p < 0.01, ***p < 0.001, and ****p < 0.0001. ns, not significant.

As mentioned above, SS enhanced the cell migration and invasion abilities compared with suspension conditions (Figure 1E–H), and A‐SSP6 cells also obtained enhanced metastatic potential through six rounds of circulation.^[^ ^14^ ^]^ We then performed Transwell assays and calculated the migrated and invaded cell numbers. The results showed that knocking down PRSS3, PAR2, and FOSL1 decreased the migration abilities of A‐SSP6 cells by 40%, 47%, and 70%, respectively (**Figure** **3**A,B). Before we observed that compared with cell migration, SS was more effective in stimulating the invasiveness of A549 cells (Figure 1E–H). Consistently, reducing the expressions of PRSS3, PAR2, and FOSL1 in A‐SSP6 cells produced a more severe inhibitory effect on the invasion ability by 80% to 90% (Figure 3C,D).

In addition to shRNAs, we also treated A‐SSP6 cells with the PRSS3 inhibitor diminazene, which has been reported previously,^[^ ^43^ ^]^ or the AP‐1 inhibitors SR11302 and T5224. The results showed that inhibiting PRSS3 or AP‐1 significantly reduced the SS survival rate, colony formation, and cell invasion abilities (Figure S7A–C, Supporting Information). In summary, decreasing the levels of PRSS3, PAR2, and FOSL1 in A‐SSP6 cells significantly reduced cell viability under SS, as well as SS‐enhanced colony formation, cell migration and invasion abilities. In particular, PRSS3, PAR2, and FOSL1 are supposed to be highly related to cell invasiveness among the four phenotypes.

Next, to examine the impacts of knocking down PRSS3, PAR2, and FOSL1 on the in vivo metastatic potential of A‐SSP6 cells, we first injected cancer cells into the tail vein of NOD/SCID mice and observed the colonies formed in the left lungs. Both fluorescent and hematoxylin and eosin (H&E) staining images presented a huge reduction in lung colonies in the knockdown groups compared with the control group. We counted the colony numbers, and the quantification results showed that reducing the expression levels of PRSS3, PAR2, and FOSL1 decreased the lung metastatic ability of A‐SSP6 cells by over 90% compared with that of control cells (Figure 3E).

We also used a lung orthotopic model that was established in our previous study to investigate the importance of PRSS3, PAR2, and FOSL1 in the spontaneous metastasis of cancer cells by injecting A‐SSP6 cells transfected with shRNAs into the right lungs of NOD/SCID mice. The body weight of each mouse was monitored for 4 weeks, and there were no significant differences in the body weights of the mice (Figure 3I). Both fluorescent images and the calculated tumor area displayed significant reductions in the primary lung tumors after knocking down PRSS3 and FOSL1, while knockdown of PAR2 had no effect on the tumorigenesis of A‐SSP6 cells (Figure 3F,G). Following this result, we examined the proliferation ability of these A‐SSP6 cells under normal culture conditions and found that similar to the primary tumor formation results, knockdown of PAR2 did not affect the proliferation rate of A‐SSP6 cells, while knockdown of the other two genes decreased cell growth (Figure S6D, Supporting Information).

Regarding metastasis, in the control group, tumor cells metastasized to the liver, intestine, brain, and skin of mice, and all eight mice in this group developed metastatic tumors. In contrast, only one or two of six or seven mice in the knockdown groups had metastatic tumors in the liver or brain (Figure 3F,H). In conclusion, based on the in vitro and in vivo results, reducing the expressions of PRSS3, PAR2, and FOSL1 significantly impaired the invasiveness and metastatic potential of A‐SSP6 cells.

### 2.5 High levels of PRSS3, PAR2, and FOSL1 Increase the Invasiveness and Metastasis of Parental Lung Cancer Cells

**Figure 4:**
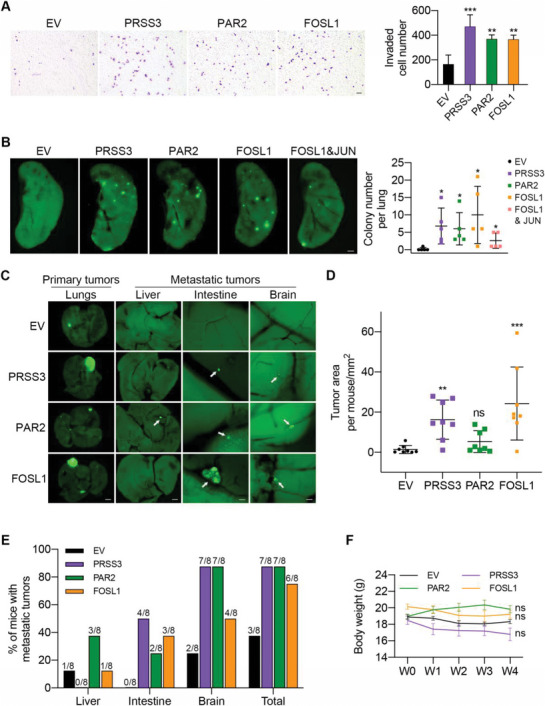
Overexpression of PRSS3, PAR2, and FOSL1 promoted invasion and metastasis of A549‐C3 cells. A) Representative images and quantification results of invaded A549‐C3 cells overexpressing PRSS3, PAR2, or FOSL1. 20 000 cells were seeded in each insert and allowed to invade for 18 h. EV, empty vector. Scale bar: 100 µm. B) Representative fluorescent images and quantification results of colonies per left lung 28 days post injection. One million A549‐C3 cells overexpressing the empty vector, PRSS3, PAR2, FOSL1 or double overexpressing FOSL1 and JUN were injected into the tail vein of each NOD/SCID mouse (*n* = 5 mice per group). Scale bar: 1 mm. C) Representative images of primary tumors in the lungs and metastatic tumors in the liver, intestine and brain (*n* = 8 mice per group). Scale bar: 2 mm (lungs and liver) and 200 µm (intestine and brain). White arrowheads indicate metastatic tumors. D) The tumor area in all five lungs of each mouse was measured by ImageJ. E) Quantified percentage of mice with metastatic tumors in each group 4 weeks after orthotopic injection was calculated. F) The body weights of each mouse at 0, 1, 2, 3, and 4 weeks after orthotopic implantation were measured. The quantification results are the means ± SD from three independent experiments or from more than five mice. Significant differences were determined by one‐way ANOVA (A,B,D) and two‐way ANOVA (F). *p < 0.05, **p < 0.01, and ***p < 0.001. ns, not significant.

To further confirm the importance of PRSS3, PAR2, and FOSL1 in promoting invasion and cancer metastasis, we overexpressed these three genes in A549‐C3 cells, which are the parental cells of A‐SSP6 cells and had low levels of PRSS3, PAR2, and FOSL1 (Figure S8A, Supporting Information). We first circulated these A549‐C3 cells for 10 and 20 h, and the results showed that overexpression of PRSS3, PAR2, and FOSL1 increased the cell viability of A549‐C3 cells, especially after 20 h of SS treatment (Figure S8B,C, Supporting Information). Elevated colony formation and cell invasion abilities were also detected in A549‐C3 cells with higher levels of PRSS3, PAR2, and FOSL1 compared with cells transfected with an empty vector (Figure S8D, Supporting Information, and **Figure** **4**A).

We then examined the lung metastatic ability after overexpression of PRSS3, PAR2, and FOSL1 and observed an increase in lung colonies in the overexpression groups (Figure 4B). In addition, considering that FRA1 and cJUN dimerize to form AP‐1, we simultaneously overexpressed FOSL1 and JUN in A549‐C3 cells and tested whether there would be more lung colonies (Figure S8A, Supporting Information). The results showed that dual overexpression of FOSL1 and JUN could not further enhance the lung metastatic ability of A549‐C3 cells, which to some extent supported our decision to focus only on FOSL1, rather than on both (Figure 4B).

To assess spontaneous metastasis after overexpression, we orthotopically injected cancer cells into the right lungs of the mice. Their body weights showed no significant differences between each experimental group and the control group (Figure 4F). Fluorescent images of the whole lungs and quantified tumor area suggested that high expression of PRSS3 and FOSL1 resulted in larger primary lung tumors, but elevated PAR2 levels did not have similar effects (Figure 4C,D). In terms of metastasis, only three of eight mice in the control group had metastatic tumors in the liver or brain. In contrast, six or seven of eight mice in the overexpression groups displayed metastases in the liver, intestine, and brain, indicating an enhanced metastatic potential of A549‐C3 cells resulting from increased PRSS3, PAR2, and FOSL1 levels (Figure 4C,E). Collectively, we can conclude that PRSS3, PAR2, and FOSL1 play important roles in the invasiveness and metastasis of cancer cells and that SS strengthens the metastatic ability of circulating cancer cells by upregulating these three genes.

### 2.6 Elevated Expression Levels of PRSS3, PAR2, and FOSL1 Are Positively Correlated with Worse Clinical Outcomes in Patients with Lung Cancer

**Figure 5:**
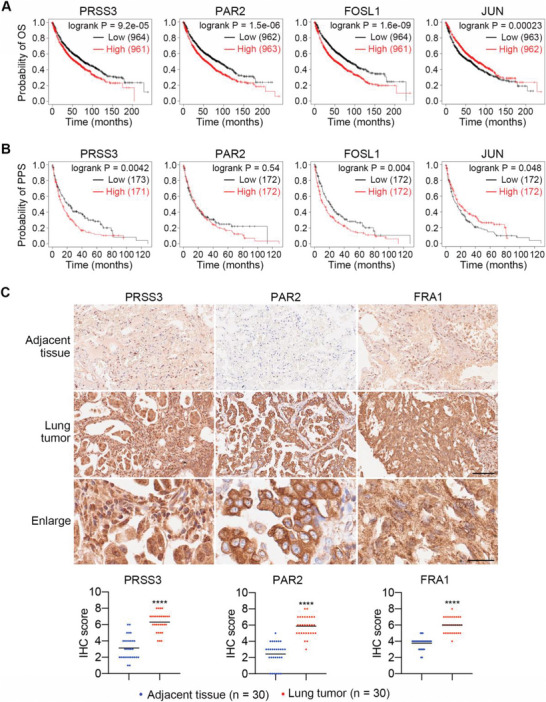
Clinical significance of PRSS3, PAR2, and FOSL1 in NSCLC. A,B) Kaplan–Meier plots of OS and PPS curves in NSCLC patients. C) Representative images and quantified IHC scores of PRSS3, PAR2, and FRA1 in adjacent tissues and lung tumors of NSCLC patients (*n* = 30). Scale bar: 100 µm (adjacent tissue and lung tumor) and 25 µm (enlarged images of lung tumors). Significant differences were determined by Student's *t*‐test (C). ****p < 0.0001.

Following the experimental evidence unveiling the important roles of PRSS3, PAR2, and FOSL1 in promoting cancer metastasis, we first investigated their clinical significance using the Kaplan–Meier plotter.^[^ ^44^ ^]^ The Kaplan–Meier plots revealed that high levels of PRSS3 and FOSL1 were linked with both shorter overall survival (OS) and post‐progression survival (PPS) of NSCLC patients, while more PAR2 only resulted in lower OS. In contrast, increased levels of JUN positively correlated with the survival of NSCLC patients (**Figure** **5**A,B). In addition, to investigate the co‐expression of these genes in clinical samples, we cross‐checked the mRNA levels of PRSS3, PAR2, and FOSL1 in lung cancer datasets using cBioPortal.^[^ ^45^, ^46^, ^47^ ^]^ The regression analysis performed by cBioPortal showed that there were positive correlations between the mRNA levels of PRSS3 versus PAR2, PRSS3 versus FOSL1, PAR2 versus FOSL1 in 230 cancer patients with lung adenocarcinoma and in 484 cancer patients with lung squamous cell carcinoma (Figure S9A,B, Supporting Information). These bioinformatic analyses provided further evidence on the clinical relevance between SS‐elevated gene expression of PRSS3, PAR2, and FOSL1 and lung cancer.

Next, we performed immunohistochemistry (IHC) staining on NSCLC patient samples to compare the levels of PRSS3, PAR2, and FRA1 between adjacent tissues (*n* = 30) and lung tumors (*n* = 30). The results showed that the IHC scores of PRSS3, PAR2, and FRA1 in lung tumors were remarkably higher than those in adjacent tissues, increasing to 2.0‐, 2.4‐, and 1.6‐fold, respectively (Figure 5C). Together, these results unveil the clinical significance of PRSS3, PAR2, and FOSL1 and suggest that they may serve as new biomarkers for the diagnosis and treatment of lung cancer metastasis.

### 2.7 PRSS3 Cleaves PAR2 and Activates the G*α*_i_‐Src‐MAPK‐FRA1/cJUN Signaling Axis to Promote the Invasion and Metastasis of Cancer Cells under SS

**Figure 6:**
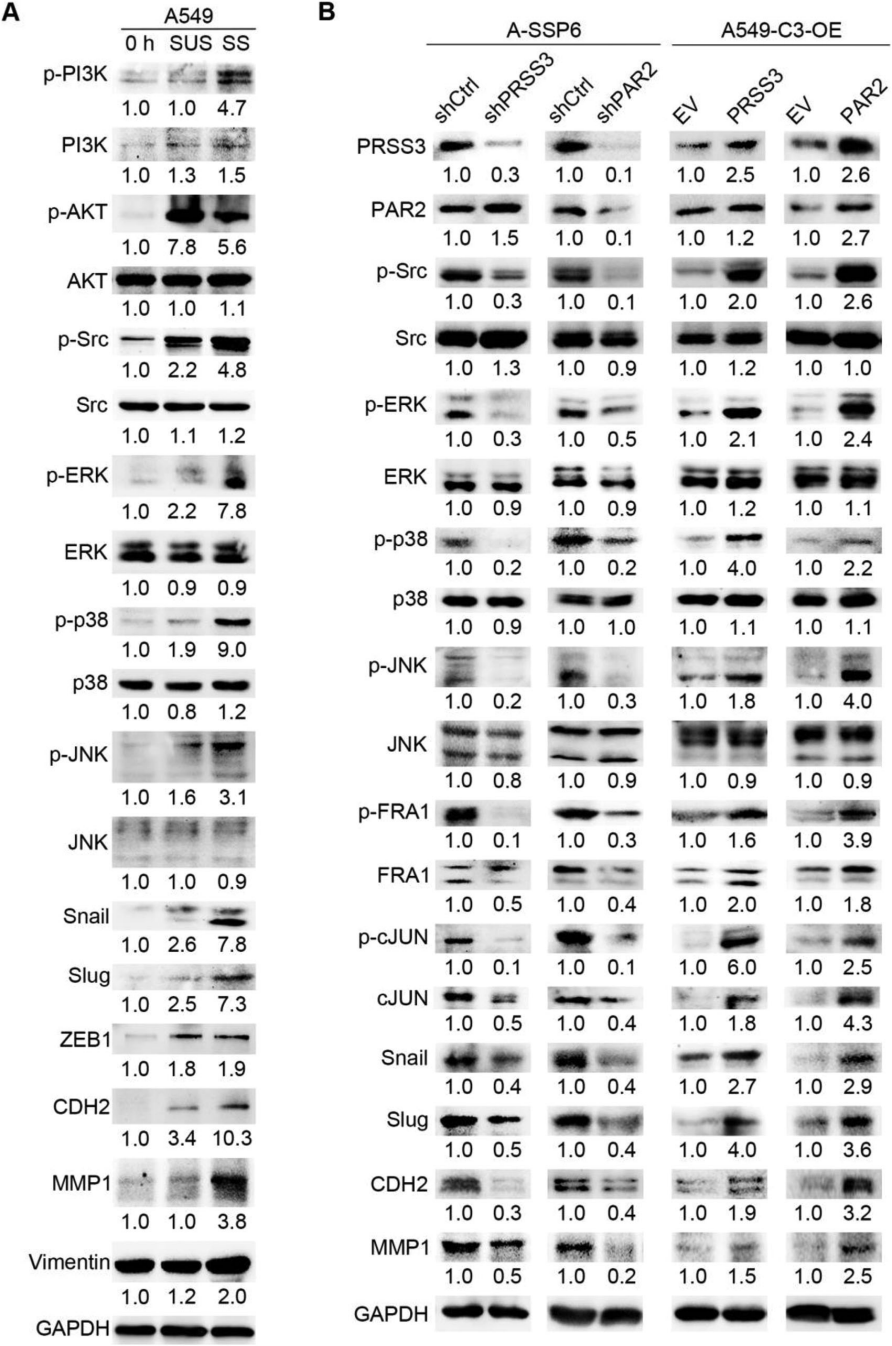
Signaling pathways induced by SS and related to PRSS3, PAR2, and FOSL1. A) Western blots showing increases in p‐Src, p‐ERK, p‐p38, p‐JNK, snail, slug, N‐cadherin (CDH2), and MMP1 in A549 cells under SS. B) Western blots showing the effects of knocking down PRSS3 and PAR2 in A‐SSP6 cells and overexpressing PRSS3 and PAR2 in A549‐C3 cells on potential downstream molecules. The quantification results are the means from three independent experiments.

To elucidate the mechanisms through which SS upregulates PRSS3, PAR2, and FOSL1 to promote cell invasion and cancer metastasis, we first examined the kinases that were reported to modulate the activity and expression of AP‐1. As the western blotting results of A549 cells under 0 h, suspension and SS conditions showed, phosphorylated PI3K was increased after circulation, but the downstream phosphorylated AKT showed no difference between suspension and SS conditions (**Figure** **6**A). In addition, treating A‐SSP6 cells with the PI3K inhibitor dactolisib did not influence the level of FRA1, indicating that PI3K‐AKT signaling was not associated with the activation of AP‐1 (Figure S10A, Supporting Information). We also detected proteins that were supposed to be upstream of AP‐1, including phosphorylated Src (p‐Src) and three mitogen‐activated protein kinases (MAPKs), phosphorylated ERK (p‐ERK), phosphorylated p38 (p‐p38), and phosphorylated cJUN N‐terminal kinase (p‐JNK). The results showed that these four kinases were significantly activated 4.8‐, 7.8‐, 9.0‐, and 3.1‐fold compared with 0 h and 2.2‐, 3.5‐, 4.7‐, and 1.9‐fold compared with suspension conditions, respectively (Figure 6A).

In addition, we investigated our RNA‐seq data and found several EMT‐related genes among the differentially expressed genes in response to SS, which could facilitate cell invasion and metastasis under the regulation of AP‐1. Then, we examined the proteins encoded by these genes using western blotting. First, the EMT‐promoting transcription factors snail and slug were increased to ≈2.5‐fold by suspension, while SS further elevated their levels to three times as high as the levels in the suspension state. In contrast, zinc finger E‐box binding homeobox 1 (ZEB1) showed no difference between the SS and suspension groups. Other mesenchymal‐promoting molecules, including N‐cadherin (CDH2), matrix metallopeptidase 1 (MMP1), and vimentin (VIM), were also elevated under SS, among which CDH2 and MMP1 showed a more significant increase (Figure 6A).

From the data presented in Figure 2B–D, we proposed that cleavage of PAR2 by PRSS3 was upstream to phosphorylate the kinases and initiate AP‐1‐related signaling pathways to promote cell invasion and cancer metastasis under SS. To validate this, we began by detecting the levels of the upregulated proteins under SS after knocking down PRSS3 and PAR2 in A‐SSP6 cells or overexpressing them in A549‐C3 cells. The results showed that reducing the expression of PRSS3 or PAR2 in A‐SSP6 cells significantly decreased the levels of p‐Src, p‐ERK, p‐p38, and p‐JNK by 50% to 80%, followed by decreases in p‐FRA1 and p‐cJUN by 90% and 70%, respectively. In addition to phosphorylated forms, the protein levels of FRA1 and cJUN were also reduced by 50% and 60%, respectively. Overexpression of PRSS3 and PAR2 in A549‐C3 cells displayed the opposite effects, which elevated the activation of the four kinases, as well as the total and phosphorylated levels of FRA1 and cJUN to 1.8‐ to 6.0‐fold (Figure 6B).

The levels of EMT‐promoting molecules, including snail, slug, CDH2, and MMP1, were also examined in the knockdown and overexpression cells. As expected, in A‐SSP6‐shPRSS3 and A‐SSP6‐shPAR2 cells, they were reduced by 50% to 80% compared with A‐SSP6‐shCtrl cells, while upregulation of PRSS3 and PAR2 led to 1.5‐ to 4.0‐fold increases in these four proteins. Moreover, we observed that the level of PAR2 was not obviously affected by the knockdown or overexpression of PRSS3, while the level of PRSS3 positively correlated with the expression of PAR2 (Figure 6B). Together, these results explain the potential mechanisms by which PRSS3 and PAR2 facilitate the invasiveness and metastatic ability of cancer cells.

**Figure 7:**
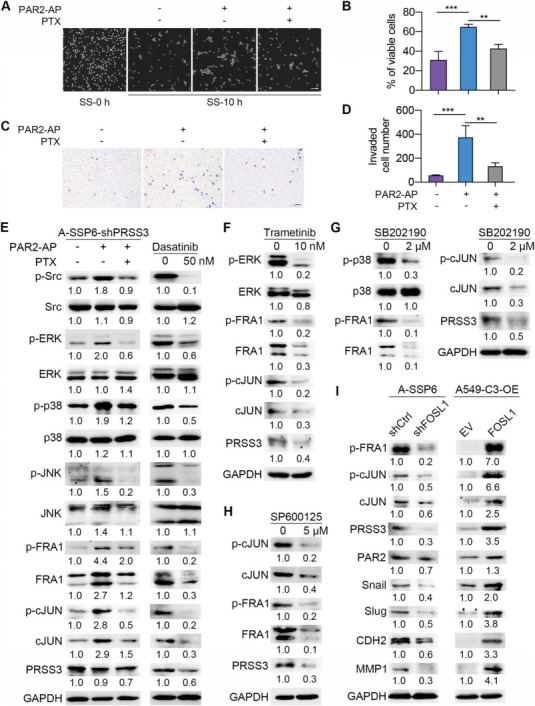
Validating the regulation of FOSL1 through the G*α*i‐Src‐ERK/p38/JNK axis and downstream molecules of FOSL1. A,B) Representative phase images and quantification results showing the percentage of viable A‐SSP6‐shPRSS3 cells after 10 h of circulation treated with 50 µm PAR2 activating peptide (PAR2‐AP) and 0.1 µg mL^−1^ G*α*i inhibitor PTX. Cells were starved in DMEM with or without adding PTX for 24 h, treated with the control peptide or PAR2‐AP for 1 h, and then cocirculated for 10 h with the indicated reagents. Scale bar: 100 µm. C,D) Representative images and quantification results of the invaded A‐SSP6‐shPRSS3 cells treated with 50 µm PAR2‐AP and 0.1 µg mL^−1^ of the G*α*i inhibitor PTX. Cells were starved in DMEM with or without adding PTX for 24 h and treated with the control peptide or PAR2‐AP for 1 h. 20 000 cells were seeded in each insert and allowed to invade for 18 h along with the treatment of PAR2‐AP and PTX as described. Scale bar: 100 µm. E) Western blots showing the changes in downstream molecules after treating A‐SSP6‐shPRSS3 cells with PTX for 24 h and PAR2‐AP for 1 h (left) and treating A‐SSP6 cells with the Src inhibitor dasatinib for 24 h (right). F–H) Western blots showing the reduction in FRA1, p‐FRA1, cJUN, p‐cJUN, and PRSS3 after treating A‐SSP6 cells with the MEK inhibitor trametinib, p38 inhibitor SB202190 and JNK inhibitor SP600125 for 24 h. I) Western blots showing the impacts of knocking down FOSL1 in A‐SSP6 cells and overexpressing FOSL1 in A549‐C3 cells on related proteins. The quantification results are the means ± SD from three independent experiments. Significant differences were determined by one‐way ANOVA (B,D). **p < 0.01, ***p < 0.001.

Next, as a GPCR, PAR2 needs to be coupled with G proteins to activate downstream signaling pathways. Since PAR2 has been reported to activate Src and MAPKs in a G*α*_i_‐dependent pathway in cancer,^[^ ^48^, ^49^ ^]^ we used the G*α*_i_ protein inhibitor pertussis toxin (PTX) to verify this under SS. Proteases cleave PAR2 and expose the SLIGKV sequence in the N‐terminus to induce the activation of this GPCR. The short peptide SLIGKV‐NH2 is synthesized as a PAR2 activating peptide (PAR2‐AP) to directly activate PAR2 without being cleaved by proteases. In A‐SSP6 cells with reduced expression of PRSS3 by shRNA, the addition of PAR2‐AP increased the cell survival rate after circulation compared with treatment with the control peptide VKGILS‐NH2, while inhibiting the G*α*_i_ protein by PTX repressed the effect of PAR2‐AP (**Figure** **7**A,B). The cell invasion ability was also influenced with a similar trend. Compared with the control peptide, PAR2‐AP significantly elevated the invaded cell number to 6.5‐fold, while PTX reduced this elevation by 65% (Figure 7C,D).

Moreover, we examined potential downstream proteins and found that PAR2‐AP increased the levels of p‐Src, p‐ERK, p‐p38, p‐JNK, p‐FRA1, FRA1, p‐cJUN, and cJUN in A‐SSP6‐shPRSS3 cells. In contrast, although PAR2‐AP could induce the activation of PAR2, inhibiting the interaction of G*α*_i_ protein with PAR2 by PTX obviously suppressed the upregulation of these proteins. The level of PRSS3 was not significantly affected, given that its mRNA level was repressed by the shRNA (Figure 7E).

Then, we treated A‐SSP6 cells with the Src inhibitor dasatinib and found that following the decrease in p‐Src, the levels of p‐ERK, p‐p38, and p‐JNK were reduced. Consequently, we observed a reduction in the protein levels of p‐FRA1, FRA1, p‐cJUN, cJUN, and PRSS3, indicating that activation of Src could regulate these proteins (Figure 7E). Afterward, we used three kinase inhibitors, including the MEK inhibitor trametinib, p38 inhibitor SB202190, and JNK inhibitor SP600125, to suppress the activation of ERK, p38, and JNK in A‐SSP6 cells. The results showed that inhibition of ERK, p38, and JNK significantly reduced the levels of p‐FRA1, FRA1, p‐cJUN, cJUN, and PRSS3, verifying the important roles of these MAPKs in modulating the activity of AP‐1 during circulation (Figure 7F–H).

Functioning as a transcription factor, AP‐1 was supposed to regulate the mRNA levels of targeted genes. Therefore, we first used qPCR to examine the changes in gene expression levels after knocking down FOSL1 in A‐SSP6 cells. The mRNA level of JUN was reduced by 70%, consistent with the self‐regulation of AP‐1 reported previously.^[^ ^50^ ^]^ The potential target genes of AP‐1, snail, slug, CDH2, MMP1, and MMP9, displayed a 40–90% reduction in transcription following the low level of FOSL1. In addition, the level of PRSS3 was decreased by 60%, while the expression of PAR2 was not affected, indicating that the transcription of PRSS3 may be modulated by AP‐1 (Figure S10B, Supporting Information). Subsequently, we detected the protein levels of these genes by western blotting. Knockdown of FOSL1 significantly reduced the protein levels of p‐FRA1, p‐cJUN, cJUN, PRSS3, snail, slug, CDH2, and MMP1 by 40% to 80%, while high expression levels of FOSL1 increased these protein levels to 2.0‐ to 7.0‐fold accordingly (Figure 7I). Upon combining the qPCR and western blotting results, we found that FOSL1 plays an important role in regulating the levels of its partner cJUN and the downstream EMT‐related proteins, as well as PRSS3.

**Figure 8:**
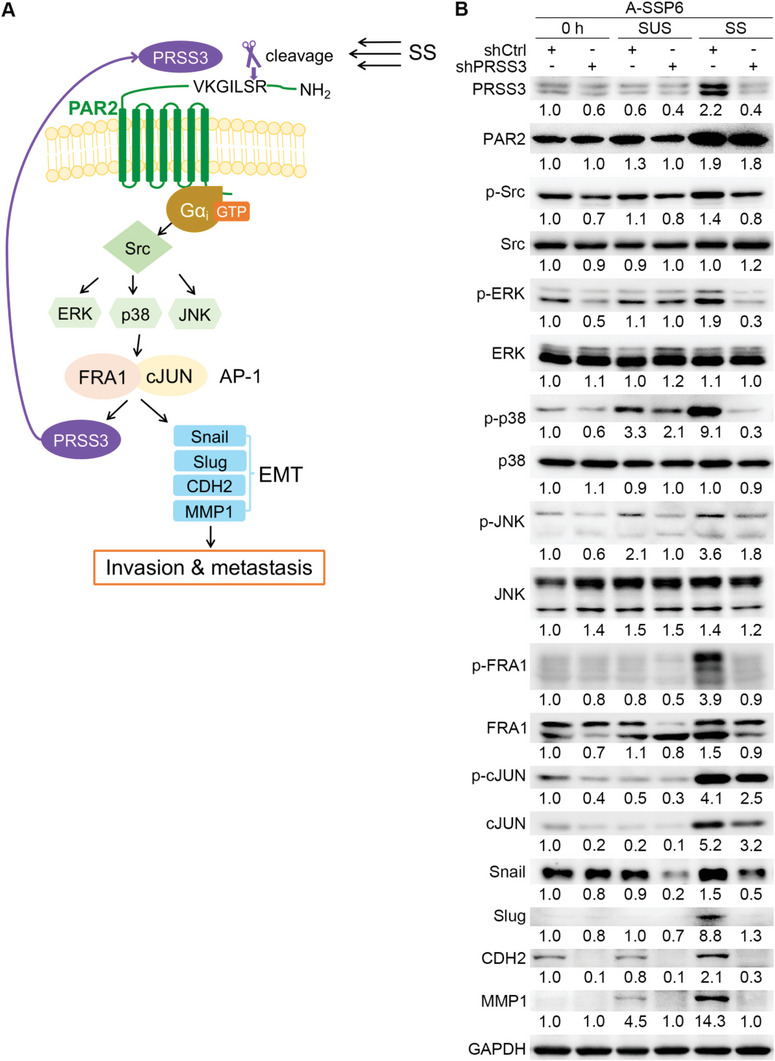
SS promotes EMT and facilitates the invasion and metastasis of cancer cells through the regulation of PRSS3, PAR2, and AP‐1. A) Proposed signaling pathways. SS first induces the cleavage activation of PAR2 by PRSS3, and then activated PAR2 is coupled with G*α*i protein to upregulate the Src‐ERK/p38/JNK‐FRA1/cJUN pathway. Under the regulation of AP‐1, EMT‐promoting molecules including snail, slug, CDH2, and MMP1 are upregulated to promote invasion and metastasis. The level of PRSS3 also increases upon the activation of AP‐1, which is then secreted out of the cells to cleave PAR2 and further enhance the signaling axis. B) Western blots showing the levels of involved molecules in A‐SSP6‐shCtrl and A‐SSP6‐shPRSS3 cells at 0 h and after 10 h of suspension and SS treatment. The quantification results are the means from three independent experiments.

Thus, we propose that PAR2 may serve as a mechanosensor during circulation. SS triggers the cleavage of PAR2 by PRSS3‐encoding mesotrypsin at an early time point, which induces the activation of this GPCR. The activated PAR2 is then coupled with G*α*_i_ protein and switches on the Src‐MAPK‐FRA1/cJUN signaling axis to facilitate the transcription of EMT‐promoting genes, including snail, slug, CDH2, and MMP1. Activation of AP‐1 also upregulates the levels of its subunits and PRSS3, which further enhances the entire process. In this way, the invasion and metastasis of circulating cancer cells are promoted by SS (**Figure** **8**A).

To better link the proposed signaling pathways with SS, we suspended and circulated A‐SSP6‐shCtrl and A‐SSP6‐shPRSS3 cells for 10 h and detected the protein levels of involved molecules. The western blotting results showed that in A‐SSP6‐shCtrl cells, the expression of PRSS3, PAR2 and downstream molecules, including p‐Src, p‐ERK, p‐p38, p‐JNK, p‐FRA1, FRA1, p‐cJUN, cJUN, snail, slug, CDH2, and MMP1, were all significantly upregulated after SS treatment compared with 0 h and suspension conditions (Figure 8B), which was consistent with the changes in A549 cells in Figures 2A and 6A. While after knocking down PRSS3 in A‐SSP6 cells, the SS‐mediated upregulations of PRSS3 and its downstream targets were significantly inhibited (Figure 8B). Although the SS‐induced increase of PAR2 was not affected by the reduction of PRSS3, SS‐mediated cleavage of PAR2 was almost completely prevented by PRSS3 inhibitor diminazene, as shown in Figure 2B. Together, these results provide the evidence that could tie our proposed PRSS3/PAR2‐triggered signaling pathways to SS.

## 3 Discussion

Both the suspension state and SS have been reported to promote the migration and invasion of cancer cells, but the distinct effects of SS on cancer cells have not been revealed.^[^ ^33^, ^51^, ^52^ ^]^ In this study, we used suspension conditions as a control to eliminate the effects of detachment and for the first time demonstrated that SS could specifically enhance the invasion ability of lung cancer cells.

The suspension state has been shown to have the potential to promote the metastasis of breast cancer cells.^[^ ^37^ ^]^ Similarly, we observed elevated migration ability of cancer cells in suspension conditions, accompanied by several hundred differentially expressed genes, indicating that the effects of detachment on CTCs should not be neglected. Subsequently, the same group reported that after SS treatment, breast cancer cells showed higher abilities to migrate and invade than adherent cells, while suspended cells migrated and invaded the most among three groups.^[^ ^51^ ^]^ The protein levels of EMT‐related N‐cadherin and snail displayed a similar trend.^[^ ^52^ ^]^ In our study, we found that SS had the strongest capacities to enhance cell migration and invasion and induced the upregulation of EMT‐related genes. This difference may be because we used a moderate level of SS (15 dyne cm^−2^) and treated cells for up to 10 h in both suspension and SS conditions, while they let cells stay static for a longer time (24 h) and circulate under low SS (4 dyne cm^−2^) for only 30 min. Consistent with our findings, another group reported an elevation of snail, slug and N‐cadherin under SS compared with the suspension state in lung cancer cells. However, cell migration was enhanced by both suspension and SS conditions compared with adherent cells and showed no significant difference between the two conditions. Most likely, a long duration of SS (72 h) and allowing cells to attach for several hours after SS treatment impaired the migration ability of circulating cancer cells.^[^ ^33^ ^]^

In our study, we found the elevation of PRSS3, PAR2, and FOSL1 to be SS‐specific effects that showed no significant increase in the suspension state. PRSS3 encodes the serine protease mesotrypsin, which belongs to the trypsin family. It was first isolated in human pancreatic tissue and fluid.^[^ ^53^ ^]^ As a minor component of pancreatic juice, PRSS3 only accounts for ≈0.5% of the secreted proteins in a normal human pancreas, while the proportions of PRSS1 and PRSS2 are 13% and 6%, respectively.^[^ ^54^ ^]^ Notably, mesotrypsin is resistant to almost all natural trypsin inhibitors, such as soybean trypsin inhibitor or human pancreatic secretory trypsin inhibitor (SPINK1).^[^ ^55^ ^]^ PRSS3 has a controversial role in cancer. Suppression of PRSS3 due to promoter methylation has been observed by several groups,^[^ ^56^, ^57^, ^58^ ^]^ while more studies have reported the pro‐cancer role of PRSS3, especially its pro‐metastatic role.^[^ ^59^, ^60^, ^61^ ^]^ In our study, we found that PRSS3 could enhance the invasion and metastatic abilities of cancer cells and was upregulated in human lung tumor tissues. Moreover, we demonstrated that SS induced the high expression of PRSS3, but not PRSS1 or PRSS2, in circulating lung cancer cells to facilitate metastasis, suggesting a crucial role of PRSS3 in metastasis‐initiating CTCs.

Finding the substrates of PRSS3 is important for determining the underlying mechanisms through which PRSS3 promotes invasion and cancer metastasis. Several inhibitors with the Kunitz domain have been identified as substrates of PRSS3,^[^ ^62^ ^]^ but none has been reported in cancer. In lung carcinoma, the mRNA level of kallikrein‐related peptidase 5 (KLK5) showed a decrease after knocking down PRSS3, and low expression of KLK5 impaired cell invasiveness.^[^ ^63^ ^]^ Since PRSS3 was able to process pro‐KLK5 to active KLK5,^[^ ^64^ ^]^ KLK5 has been proposed as a potential substrate of PRSS3. In our RNA‐seq data, PAR2, but not KLK5, was increased after SS treatment. Further experiments displayed the pro‐metastatic ability of PAR2, and PAR2 was identified as a potential target for PRSS3 during circulation.

PARs are a group of GPCRs, and PAR2 is more efficiently cleaved and activated by trypsin.^[^ ^65^ ^]^ Activation of PAR2 by trypsin, which usually refers to trypsin 1/2 encoded by PRSS1/2, was confirmed by measuring cellular calcium concentrations in cancer.^[^ ^66^, ^67^ ^]^ The association of PRSS3 with PAR2 has been presented in esophageal adenocarcinoma to promote cell proliferation and survival.^[^ ^65^ ^]^ In addition, trypsin IV is a splice variant of mesotrypsin, and its cleavage of PAR2 was shown by high‐performance liquid chromatography and subsequent mass spectrometry analysis.^[^ ^42^, ^68^ ^]^ In this study, we inserted a red fluorescent protein before the trypsin cleavage site in PAR2 to visualize cleavage by PRSS3 under SS following previous descriptions.^[^ ^39^ ^]^ We reported for the first time the SS‐specific cleavage of PAR2 by PRSS3 in circulating cancer cells. In addition, this cleavage was not able to occur when there was a mutation at the cleavage site, and inhibiting PRSS3 also suppressed the cleavage, further proving the potential of PAR2 to be the substrate of PRSS3. More importantly, we observed that PAR2 was cleaved within 2 h of SS treatment, indicating that PAR2 activation is an early response to SS. Therefore, we propose that PAR2 may serve as a mechanosensor in circulation to enhance the metastatic ability of CTCs.

PAR2 activation by trypsin or PAR2‐AP could facilitate cancer progression through G protein‐ or *β*‐arrestin‐dependent pathways.^[^ ^48^, ^49^, ^69^ ^]^ Both *β*‐arrestin 1 and 2 were significantly decreased by 80% and 50% respectively after circulatory treatment, while the G*α*_i_ protein inhibitor suppressed the invasiveness and downstream molecules activated by PAR2‐AP in A‐SSP6‐shPRSS3 cells, indicating that SS promoted cell invasion and metastasis through a G*α*_i_‐dependent signaling pathway. Subsequently, the Src‐MAPK‐FRA1/cJUN signaling axis was activated under SS, which was also stimulated in previous studies investigating how PAR2 activation enhanced cancer progression.^[^ ^48^, ^49^, ^67^, ^70^, ^71^ ^]^

Misregulation of the transcription factor AP‐1 has been reported in different types of cancers.^[^ ^72^ ^]^ It is composed of the dimerization of subunits, which are proteins from the Fos and Jun families in most cases. Jun proteins can form homodimers by themselves, while Fos proteins have to bind to Jun proteins to form heterodimers. AP‐1 is known to respond to various kinds of stress, including SS. SS induced activation and upregulation of FOS from the Fos family and cJUN from the Jun family, as well as higher DNA binding ability of AP‐1, in adherent endothelial cells and nucleus pulposus cells.^[^ ^73^, ^74^ ^]^ In primary human osteoblasts and murine MC3T3‐E1 cells, fluid SS increased the mRNA levels of Fos family members, including FOS, FOSL1, FOSL2, and FOSB.^[^ ^75^ ^]^ In cancer, only one study has reported that FOSL1 and JUN were upregulated transcriptionally to 3.7‐ and 2.0‐fold, respectively, in adherent colon cancer cells after experiencing a 15 dyne cm^−2^ shear flow for 12 h.^[^ ^76^ ^]^ To our knowledge, we are the first to display the activation, nuclear translocation, and upregulation of FRA1 and cJUN induced by SS in cancer cells. Our data showed that high levels of activated FRA1 and cJUN were detected in the nucleus in the early response to SS and augmented the transcription of target genes to facilitate metastasis. In addition to the phosphorylated forms, we also found that both mRNA and protein levels of FOSL1 and JUN were increased after SS treatment, consistent with the self‐regulation of AP‐1 reported previously.^[^ ^50^ ^]^

In the present study, we mainly focused on FOSL1 from the Fos family due to its high expression under SS. The important role of FOSL1 in promoting cancer metastasis has been revealed in various cancers.^[^ ^72^, ^77^, ^78^ ^]^ Consistently, we showed that knockdown of FOSL1 significantly attenuated the SS survival rate, as well as the migration, invasion and colony formation abilities of SS‐resistant A‐SSP6 cells. Fewer lung colonies and metastases were observed in tail vein injection and orthotopic mouse models, respectively. In addition, overexpression of FOSL1 enhanced the metastatic ability of parental A549‐C3 cells. Interestingly, when we elevated the levels of FOSL1 and JUN simultaneously, the lung metastatic ability of cancer cells did not increase compared with cells that only had FOSL1 overexpression. This may be explained by the elevation of cJUN and p‐cJUN in cells overexpressing FOSL1. Moreover, Fos‐Jun heterodimers showed a higher affinity and activity to bind to DNA than Jun homodimers,^[^ ^79^, ^80^ ^]^ and our results suggest a unique role of FRA1 in forming AP‐1 with cJUN and promoting SS‐induced cancer metastasis.

Studies using microfluidic systems to mimic blood SS, including ours, all have the limitation of not fully representing the in vivo situations that CTCs encounter. It could be improved to some extent if other factors are included in the circulatory system, such as platelets, immune cells, or endothelial cells. However, SS‐induced EMT and enhanced invasion ability were also observed in CTCs isolated from human or mouse blood.^[^ ^7^, ^81^, ^82^, ^83^ ^]^ Upregulation of FOS and JUN in CTCs compared with primary tumors in a lung tumor mouse model further supported the important role of AP‐1 in circulation,^[^ ^84^ ^]^ suggesting the potential of our findings in the microfluidic system to be applied in clinical trials.

Previous studies have revealed the crucial role of cytoskeleton rearrangement in SS‐enhanced cell migration and invasion.^[^ ^26^, ^30^, ^32^, ^51^, ^85^ ^]^ Reorganization of F‐actin, accompanied by the upregulation of members of the Rho GTPase family, including RhoA, Rac1, and cell division control protein 42 (Cdc42), was observed in these studies. In our study, we did not focus on cytoskeletal changes in suspension or SS conditions. However, the mRNA levels of RhoA, RhoB, RhoC, Rac1, and Cdc42 were increased in the SS group according to our RNA‐seq results, suggesting the involvement of actin reorganization during circulation. In addition, elevated expression levels of several tubulin‐related genes were also found in our RNA‐seq data. For instance, tubulin alpha‐4a (TUBA4A), which encodes an alpha tubulin, increased to 13.5‐fold under SS compared with suspension conditions. Therefore, it would be interesting to investigate how the cytoskeleton is changed during SS and determine the function of these changes. In addition, one group observed autophagy induced by SS in adherent liver cancer cells, which accounts for the enhanced migration and invasion abilities after SS treatment.^[^ ^85^ ^]^ In our data, the autophagy markers mentioned in these studies showed no significant changes after SS treatment.

In summary, our results unveil the distinct pro‐metastatic effects of SS on cancer cells, excluding the impacts of detachment. Among the genes that were particularly upregulated by SS, PRSS3, PAR2, and FOSL1 played important roles in cell invasion and cancer metastasis. SS first induces the cleavage of PAR2 by PRSS3/mesotrypsin, which activates the GPCR. Then, activation of PAR2 stimulates the Src‐MAPK‐FRA1/cJUN signaling axis in a G*α*_i_‐dependent manner to upregulate EMT‐related molecules to promote the invasion and metastasis of circulating cancer cells. Moreover, higher levels of PRSS3, PAR2, and FOSL1 were observed in lung tumors and were linked with worse clinical outcomes in NSCLC patients. Our findings identified PRSS3, PAR2, and FOSL1 as SS‐responsive genes and suggest the potential of PAR2 as a mechanosensor for distinguishing metastasis‐initiating CTCs and developing new therapies to prevent and treat metastasis.

## 4 Experimental Section

### Cell Lines and Cell Culture

The NSCLC cell line A549, the breast cancer cell lines MDA‐MB‐231 and MDA‐MB‐468, the colorectal cancer cell line HCT‐116, and the human embryonic kidney cell line 293T were obtained from the American Type Culture Collection (ATCC) and cultured in Dulbecco's modified Eagle's medium (DMEM; #12100046, Thermo Fisher Scientific, USA). The NSCLC cell line H1975 was obtained from Prof. Joong Sup Shim at the Faculty of Health Sciences at the University of Macau, Macau, China and cultured in Roswell Park Memorial Institute 1640 medium (#31800022, Thermo Fisher Scientific, USA). All culture media were supplemented with 10% fetal bovine serum (#10270‐106, Gibco, USA) and 1% penicillin–streptomycin antibiotics (#15140122, Thermo Fisher Scientific, USA). A549‐C3 and A‐SSP6 cells were generated as previously described.^[^ ^14^ ^]^

### Reagents

The PRSS3 inhibitor diminazene (#18678‐1) was obtained from Cayman Chemical (USA). The AP‐1 inhibitor T5224 (#S8966), MEK inhibitor trametinib (#S2673), and PI3K inhibitor dactolisib (#S1009) were purchased from Selleckchem (USA). The JNK inhibitor SP600125 (#420119) and p38 inhibitor SB202190 (#559388) were obtained from Calbiochem (Germany). G*α*_i_ protein inhibitor PTX (#sc‐200837) was purchased from Santa Cruz (USA). Another AP‐1 inhibitor SR11302 (#2476), PAR2‐AP (SLIGKV‐NH2; #3010), and the control peptide (VKGILS‐NH2; #3392) were obtained from Tocris Bioscience (UK).

### Suspension Conditions and Microfluidic Circulatory System

Cancer cells were trypsinized, resuspended, and seeded at a density of 2 × 10^5^ cells per mL in normal culture medium into an ultralow attachment 6‐well plate (#3471, Corning, USA). Cells were incubated in a CO_2_ incubator at 37 °C for the indicated durations and phase images were taken using a Carl Zeiss microscope (10× objective, Axio Observer, Germany).

The microfluidic circulatory system was developed as described in the authors’ previous studies.^[^ ^11^, ^14^, ^22^, ^23^ ^]^ In short, the system was composed of a reservoir with a cotton filter on top to avoid contamination and evaporation of culture medium, a connecting tube, a peristaltic pump (Ismatec, Germany) for generating a pulsatile shear flow, and a circulatory tube 500 µm in diameter and 1.5 m in length. The level of SS could be calculated using the Poiseuille's equation *τ* = $4Q\eta /\pi R^{3}$, where *τ* was the calculated SS level in dyne cm^−2^, *Q* was the flow rate in cm^3^ s^−1^, *η* was the dynamic viscosity of the fluid (0.012 dyne s cm^−2^), and *R* was the radius of the tubes (250 µm). In this study, the focus was mainly on the effects of SS15 since it represented the average SS in human arteries.^[^ ^19^ ^]^

All tubes were sterilized with 70% ethanol and washed with Milli‐Q water three times. To prevent cells from adhering to the tubes, the whole system was then coated with 1% Pluronic F‐127 (#P2443, Sigma‐Aldrich, Germany) before use. Cancer cells were trypsinized and resuspended at a density of 2 × 10^5^ cells per mL in normal culture medium. 1 mL of the cell suspension was added into the reservoir and circulated under the indicated conditions. After circulatory treatment, cells were collected and added into a confocal dish for imaging with a Carl Zeiss microscope (10× objective, Axio Observer, Germany).

### MTT Assay

Cells were collected from 0 h, suspension, and SS conditions, and 100 µL of cells was seeded in 96‐well plates. After incubation with 10 µL of MTT (#M2128, Sigma‐Aldrich, Germany) solution for 4 h, 100 µL of 10% SDS with 0.01 m HCl was added to each well and incubated overnight. The absorbance was measured at 595 nm using a plate reader (PerkinElmer VICTOR X3, USA).

### Transwell Migration and Invasion Assays

Cells were collected after suspension and SS treatment or trypsinized and then resuspended in DMEM with no serum. 10 000 cells (5000 for MDA‐MB‐231 and MDA‐MB‐468 cells), with a volume of 100 µL, were seeded on the membrane of the Transwell chamber (#3422, Corning, USA) and 600 µL of normal culture medium was added to the lower chamber. After incubation in the cell culture incubator, cells on the top side of the membrane were wiped off with a cotton swab and the migrated cells on the bottom side were fixed with 4% paraformaldehyde (PFA; #158127, Sigma‐Aldrich, Germany) for 15 min and stained with 0.5% crystal violet (#C6158, Sigma‐Aldrich, Germany) for another 15 min. Afterward, the membrane was cut off and mounted on a glass slide using mounting medium (#06522, Sigma‐Aldrich, Germany).

In the invasion assay, the Transwell membrane was first coated with 100 µL of Matrigel (#356230, Corning, USA) at 37 °C for 2–3 h, which was diluted with DMEM containing no serum at a ratio of 1–30. Then, the cells were collected or trypsinized and seeded similarly, but at a density of 2 × 10^5^ cells per mL (1 × 10^5^ cells per mL for MDA‐MB‐231 and MDA‐MB‐468 cells). Images were taken using a bright‐field microscope (M165 FC, Leica, Germany), and all the cells on the membrane were counted.

### Colony Formation Assay

1000 cells per well (2000 for H1975 cells and 5000 for MDA‐MB‐468 cells) were seeded in 6‐well plates and cultured in normal medium for 10 days (7 days for A‐SSP6 cells). The medium was then removed, and the cells were stained with 0.5% crystal violet for 15 min. Images of the colonies were taken using a digital camera and the colony number or area of each well was measured using ImageJ.

### RNA Sequencing Analysis

A549 cells at 0 h or suspended or circulated under SS15 for 10 h were collected and resuspended in TRIzol (#15596026, Thermo Fisher Scientific, USA). Then, the samples were sent to Novogene (China) for RNA sequencing analysis.

### RNA Extraction and qPCR

Cells were dissolved in TRIzol, and total RNA was extracted. Reverse transcription was conducted using an iScript cDNA synthesis kit (#1778890, Bio‐Rad, USA). qPCR was then performed using iTaq Universal SYBR Green (#1725122, Bio‐Rad, USA). The primers used are listed in Table S1, Supporting Information.

### Western Blot Analysis

Cells were collected, washed with phosphate‐buffered saline (PBS) once, and lysed in radioimmunoprecipitation assay buffer supplemented with protease inhibitor and phosphatase inhibitor cocktail (Sigma‐Aldrich, Germany). The protein concentrations were determined by the Bio‐Rad protein assay. The same amounts of proteins were loaded in SDS–polyacrylamide gels, separated via electrophoresis, and transferred to a nitrocellulose membrane (Bio‐Rad, USA). The membrane was blocked with 5% blotting‐grade blocker (#1706404, Bio‐Rad, USA) and probed with primary antibodies overnight at 4 °C. The next day, the blots were washed and probed with HRP‐conjugated secondary antibodies for 1 h at room temperature. Detailed information about the primary and secondary antibodies is listed in Table S2, Supporting Information. The blots were finally incubated in Clarity Western ECL Substrate (#1705061, Bio‐Rad, USA) and visualized using a ChemiDoc Touch Imaging System (Bio‐Rad, USA). The intensity of each band was measured by ImageJ and the significance of WB results was confirmed through Student's *t*‐test.

### Immunofluorescence Staining

Sterilized glass coverslips were precoated with poly‐d‐lysine hydrobromide (#P7886, Sigma‐Aldrich, Germany) for 30 min to help cells adhere on coverslips. Cancer cells were collected and seeded onto these coverslips and incubated at 37 °C for 20–30 min. Then, the cells were fixed with 4% PFA for 15 min, permeabilized with 0.2% Triton X‐100 (#T8787, Sigma‐Aldrich, Germany) for 15 min, and blocked with 3% bovine serum albumin (#4240GR500, BioFroxx, Germany) for 1 h. Next, the cells were incubated with primary antibodies overnight at 4 °C and with Alexa fluorescent‐conjugated secondary antibodies (Invitrogen, USA) for 1 h at room temperature. The nuclei were labeled with Hoechst 33342 (#H3570, Thermo Fisher Scientific, USA), and fluorescent images were captured using a confocal microscope (Carl Zeiss Confocal LSM710, Germany). Fluorescence intensity was measured by ImageJ.

### Gene Knockdown and Overexpression (Including N‐mCherry‐PAR2, With or Without R36A)

Sequences of shRNAs were determined by calculating the H‐b index of the candidates to obtain better knockdown efficiencies.^[^ ^86^ ^]^ The targeting sequences are shown in Table S3, Supporting Information, and the overexpression sequences are shown in Table S4, Supporting Information.

In addition to normal overexpression, the red fluorescent protein mCherry was inserted before the cleavage site of the PAR2 protein to observe cleavage according to previous descriptions^[^ ^39^, ^40^ ^]^ and transfected the plasmid into A549 cells. Red fluorescence should exist on the cell membrane if cleavage does not occur. In addition, a mutation of arginine to alanine was made at the cleavage site for trypsin so that PAR2 could not be cleaved and activated. All shRNA and overexpression vectors were purchased from VectorBuilder Company (USA).

### Lung Colony Formation Assay and Orthotopic Lung Xenografts

All animal experiments were approved by the University of Macau Animal Ethics Committee (Approved Protocol ID: UMARE‐025‐2017 and UMARE‐026‐2017). For the lung colony formation assay, 1 × 10^6^ cells were injected into the tail vein of 6‐ to 8‐week‐old female NOD/SCID mice. Mice were sacrificed 28 days post injection, and lung tissues were dissected for imaging with an Olympus fluorescence microscope (MVX10, Japan). Lung colonies on the left lungs of mice were counted to evaluate the lung metastatic ability of cancer cells.

The method of generating spontaneous lung tumors and observing metastasis had been described previously.^[^ ^14^ ^]^ Cancer cells were resuspended at a density of four million cells per 25 µL in PBS and mixed with 25 µL of Matrigel matrix (#354234, Corning, USA). NOD/SCID mice were anesthetized with 1.25% avertin (#T4840‐2, Sigma‐Aldrich, Germany), and then 50 µL of the mixture was injected into the right lungs of each mouse by a 30‐gauge hypodermic needle. The mice were weighed every week. 4 weeks post injection, mice were sacrificed, and tissues were imaged by the Olympus fluorescence microscope (MVX10, Japan).

### Immunohistochemistry

For H&E staining, the left lungs of mice were fixed with 4% PFA, dehydrated, embedded in paraffin, and cut into 5‐µm‐thick sections. The tissue sections were deparaffinized and stained with H&E using a Leica ST5020‐CV5030 Multistainer‐Coverslipper (Germany).

Tissue microarray slides of NSCLC patients were obtained from Superchip Company (China). After deparaffinization and antigen retrieval, IHC was performed using the IHC Detection Kit (#ab64264, Abcam, UK) according to the manufacturer's instructions. Nuclei were stained with hematoxylin by a Leica ST5020‐CV5030 Multistainer‐Coverslipper (Germany). All slides were scanned with a NanoZoomer S60 Digital slide scanner (#C13210‐01, HAMAMATSU, Japan) to acquire whole‐view images. The IHC scores were composed of percentage scores and intensity scores as previously described.^[^ ^87^ ^]^

### Statistical Analysis

All data were acquired from at least three independent experiments or more than five mice and were presented as the means ± SD. Statistical significance was determined by one‐way analysis of variance (ANOVA), two‐way ANOVA, or Student's *t*‐test using GraphPad Prism 9.0. *p < 0.05, **p < 0.01, ***p < 0.001, and ****p < 0.0001 were considered statistically significant.

## Conflict of Interest

The authors declare no conflict of interest.

## Author Contributions

M.Z. and K.Q.L. designed the research studies. M.Z. and K.L. conducted the experiments. M.Z. and K.Q.L. analyzed the data. K.Q.L. and M.Z. wrote the manuscript.

## Supporting information

Supporting Information

## Data Availability

The data that support the findings of this study are available from the corresponding author upon reasonable request.
