# Supplementary material for: Shear Stress Drives the Cleavage Activation of Protease‐Activated Receptor 2 by PRSS3/Mesotrypsin to Promote Invasion and Metastasis of Circulating Lung Cancer Cells
Source: Adv Sci (Weinh). 2023 Jul 3;10(25):2301059. doi: 10.1002/advs.202301059 (PMC10477893; doi:10.1002/advs.202301059)
Supplement: Supplementary file 1 — Supporting Information [file ADVS-10-2301059-s001.pdf]

ADVANCED SCIENCE
Open Access

Supporting Information

for *Adv. Sci.*, DOI 10.1002/advs.202301059

Shear Stress Drives the Cleavage Activation of Protease-Activated Receptor 2 by PRSS3/Mesotrypsin to Promote Invasion and Metastasis of Circulating Lung Cancer Cells

*Muya Zhou, Koukou Li and Kathy Qian Luo**

Supporting Information

Shear stress drives the cleavage activation of protease-activated receptor 2 by PRSS3/mesotrypsin to promote invasion and metastasis of circulating lung cancer cells

Muya Zhou[1], Koukou Li[1], Kathy Q. Luo[1, 2]*

Supplementary figures

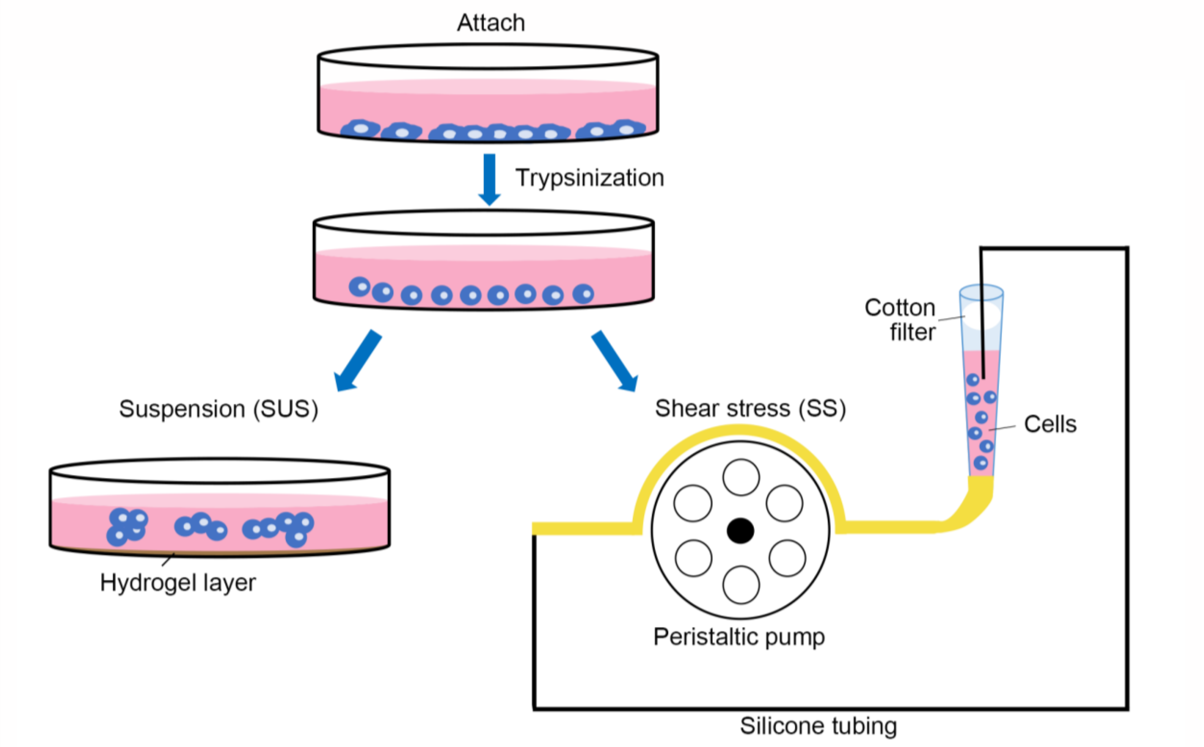

Figure S1. Schematic diagram of the experimental design. Attached cells were trypsinized and seeded on hydrogel-coated ultralow attachment 6-well plates for suspension conditions or

added to a microfluidic circulatory system to undergo SS.

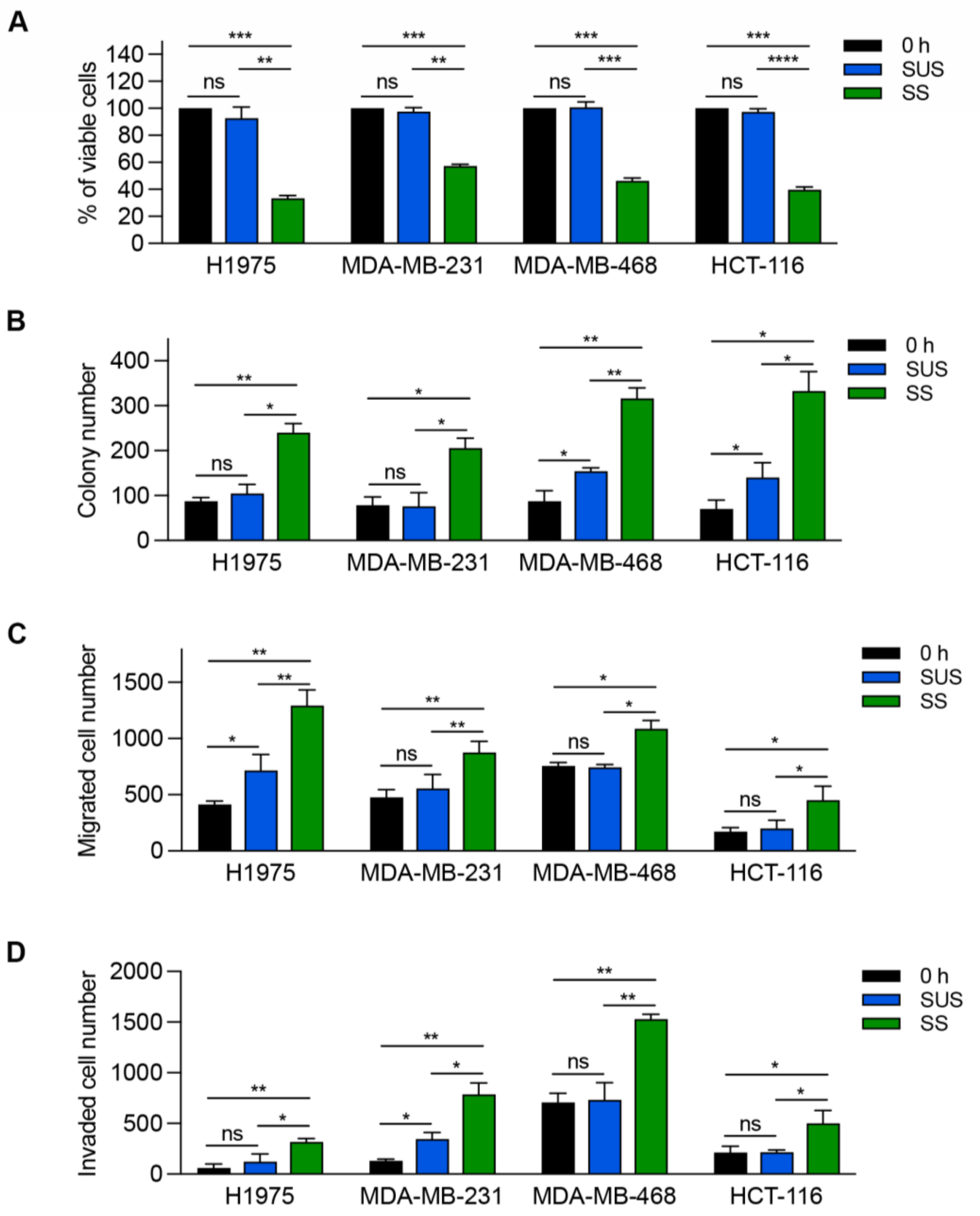

Figure S2. SS induced cell death and enhanced migration, invasion and colony formation in other cancer cell lines. (**A**) Quantified cell viability of H1975, MDA-MB-231, MDA-MB-468 and HCT116 cells at 0 h and after 10 h of suspension and SS treatment. (**B** to **D**) Quantification results of the colony formation, Transwell migration and invasion abilities of H1975, MDA-MB-231, MDA-MB-468 and HCT116 cells under the indicated conditions. For the colony formation assay, 2×10^3 H1975 cells, 1×10^3 MDA-MB-231 cells, 5×10^3 MDA-MB-468 cells and 1×10^3 HCT116 cells were seeded in each well of 6-well plates and allowed to grow for 10 days. Ten thousand H1975 and HCT116 cells, and five thousand MDA-MB-231 and MDA-MB-468 cells were seeded in the Transwell migration assay. For the invasion assay, 2×10^4 H1975 and HCT116 cells, and 1×10^4 MDA-MB-231 and MDA-MB-468 cells were seeded. Cells were then allowed to migrate or invade for 18 h. The quantification results are

the means ± SD from three independent experiments. Significant differences were determined by two-way ANOVA. *P < 0.05, **P < 0.01, ***P < 0.001 and ****P < 0.0001. ns, not significant.

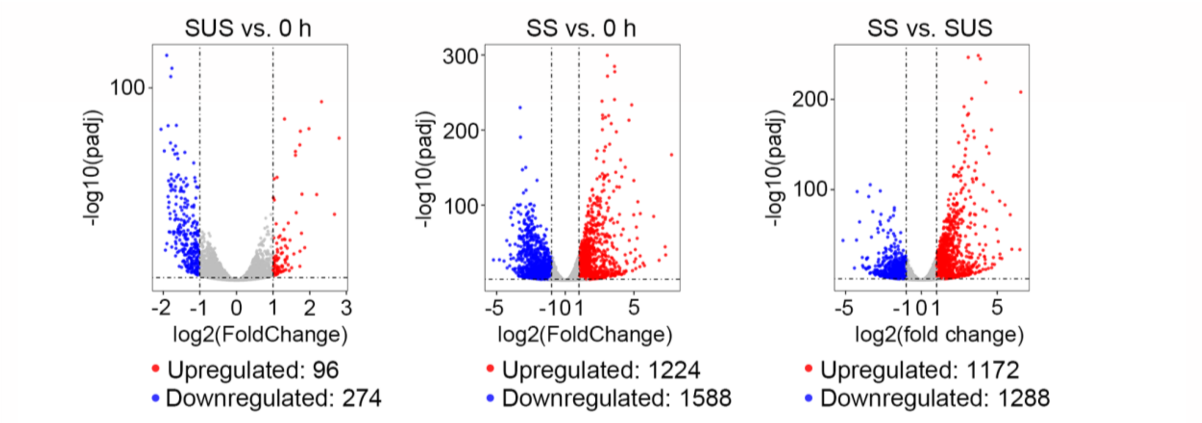

Figure S3. Volcano plots showing the differentially expressed genes in SUS vs. 0 h, SS vs. 0 h and SS vs. SUS. The thresholds were set as P < 0.05, a fold change ≥ 2 for upregulated genes and a fold change ≤ 0.5 for downregulated genes.

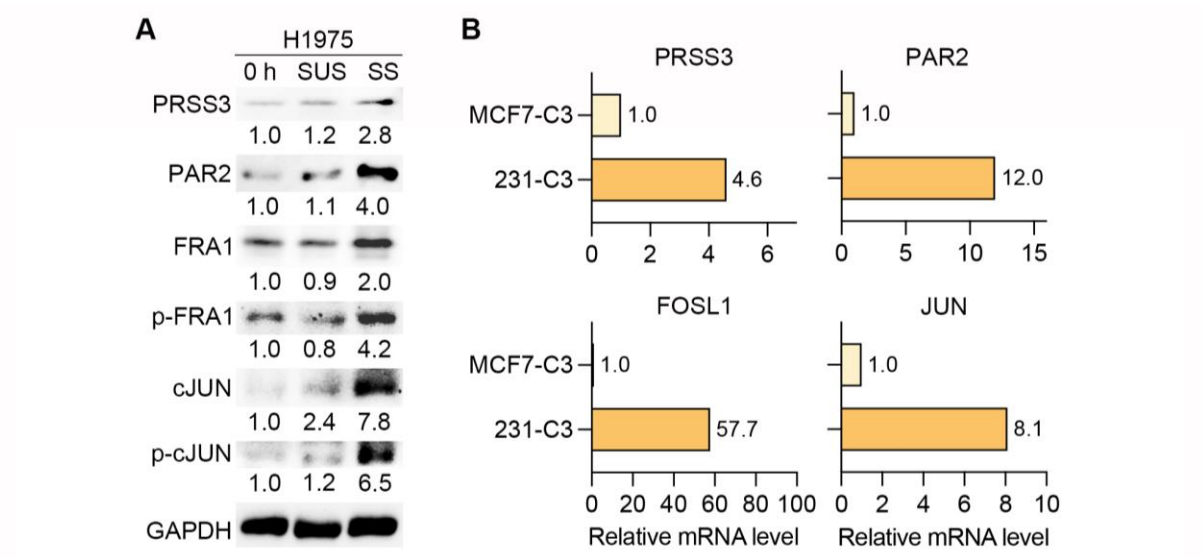

Figure S4. The levels of PRSS3, PAR2, FOSL1 and JUN were increased by SS in another NSCLC cell line H1975 and in more metastatic breast cancer cells. (**A**) Western blots showing the upregulation in protein levels of PRSS3, PAR2, FRA1, p-FRA1, cJUN and p-cJUN after SS compared with both 0 h and suspension conditions in H1975 cells. (**B**) Relative mRNA levels of PRSS3, PAR2, FOSL1 and JUN in 231-C3 cells compared with MCF7-C3 cells based on RNA-seq analysis. The quantification results are the means from three independent experiments.

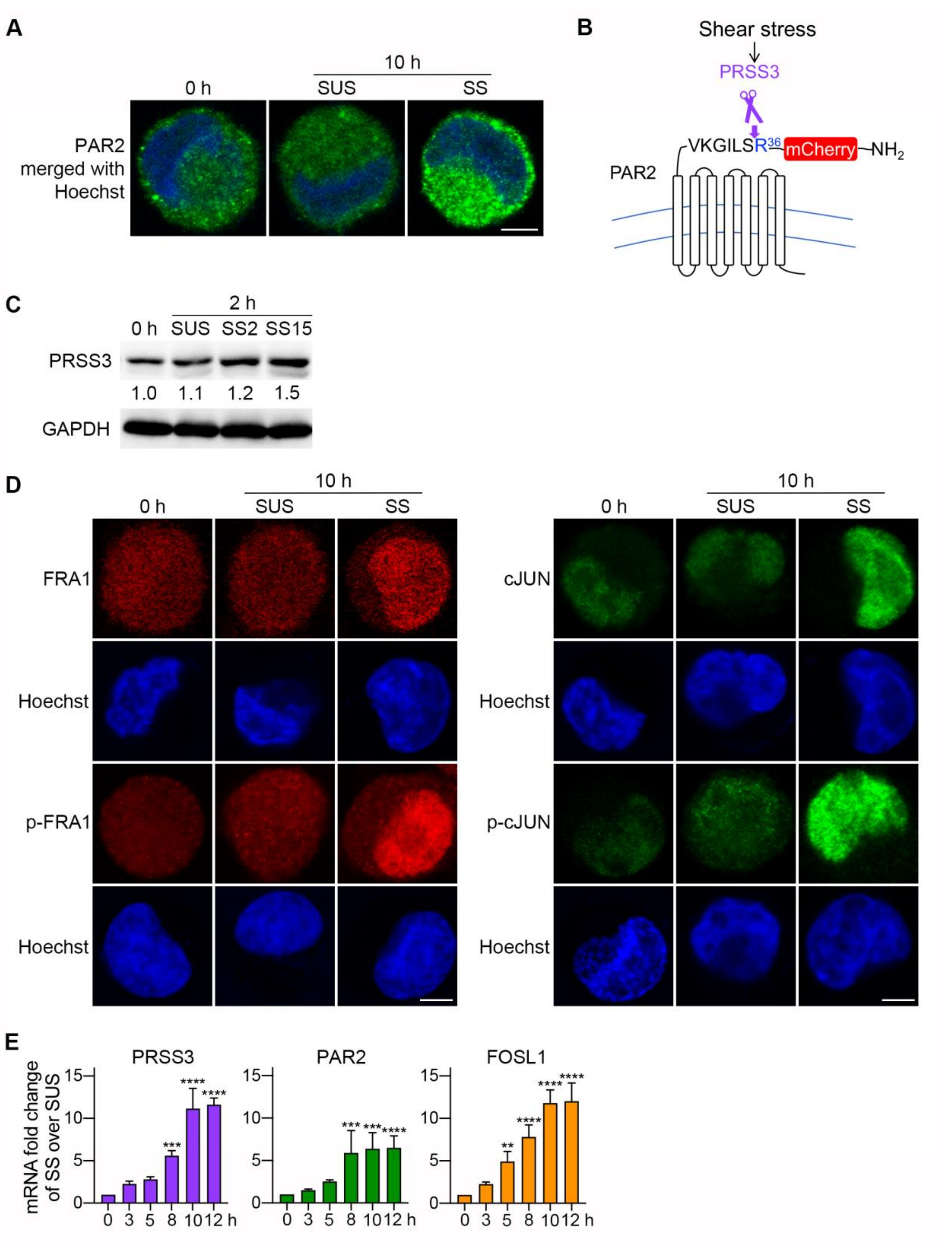

Figure S5. Increases of PRSS3, PAR2, FOSL1 and JUN induced by SS were well validated. (**A**) Representative IF staining images of PAR2 in A549 cells under the indicated conditions. Scale bar, 5 μm. (**B**) Schematic showing that the fluorescent mCherry sequence was inserted into the N-terminus of PAR2 before the PRSS3 cleavage site. (**C**) Western blots

showing the levels of PRSS3 in A549 cells at 0 h and after 2 h of suspension, SS2 and SS15 treatment. (**D**) Representative IF staining images showing the subcellular localization of FRA1, p-FRA1, cJUN and p-cJUN before and after 10 h of suspension and SS treatment. Scale bar, 5 μm. (**E**) qPCR results showing the relative mRNA fold changes of PRSS3, PAR2 and FOSL1 in SS versus suspension after 0, 3, 5, 8, 10 and 12 h of treatment. The quantification results are the means ± SD from three independent experiments. The statistical analysis was performed compared with the value of 0 h and significant differences were determined by two-way ANOVA (E). $^{**}P < 0.01$, $^{***}P < 0.001$ and $^{****}P < 0.0001$.

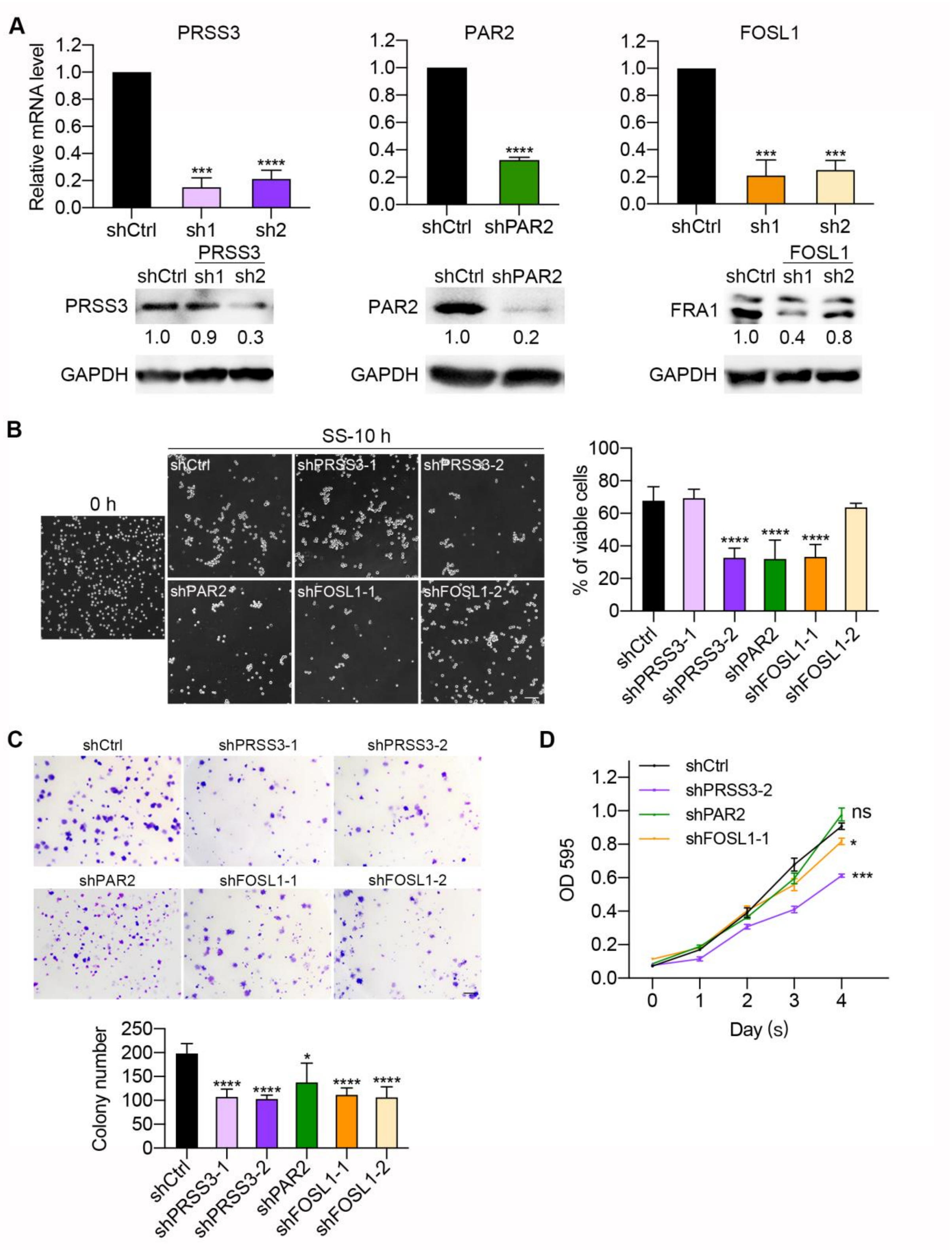

Figure S6. Knockdown of PRSS3, PAR2 and FOSL1 reduced the SS survival rate and colony formation of A-SSP6 cells. (**A**) Knockdown efficiencies of PRSS3, PAR2 and FOSL1 in A-SSP6 cells validated by qPCR (upper) and Western blotting (lower). (**B**) Representative phase images and quantified percentage of viable A-SSP6 cells transfected with shRNAs

before and after 10 h of circulation. Scale bar, 100 µm. (**C**) Representative images and quantified number of colonies formed by A-SSP6 cells after knocking down PRSS3, PAR2 and FOSL1. One thousand cells were seeded and allowed to grow for 7 days. Scale bar, 2 mm. (**D**) Growth curves of transfected A-SSP6 cells as determined by the MTT assay. The quantification results are the means ± SD from three independent experiments. Significant differences were determined by one-way ANOVA (A to C) and two-way ANOVA (D), except for the knockdown efficiency of PAR2 which was determined by Student's *t* test. **P* < 0.05, ****P* < 0.001 and *****P* < 0.0001. ns, not significant.

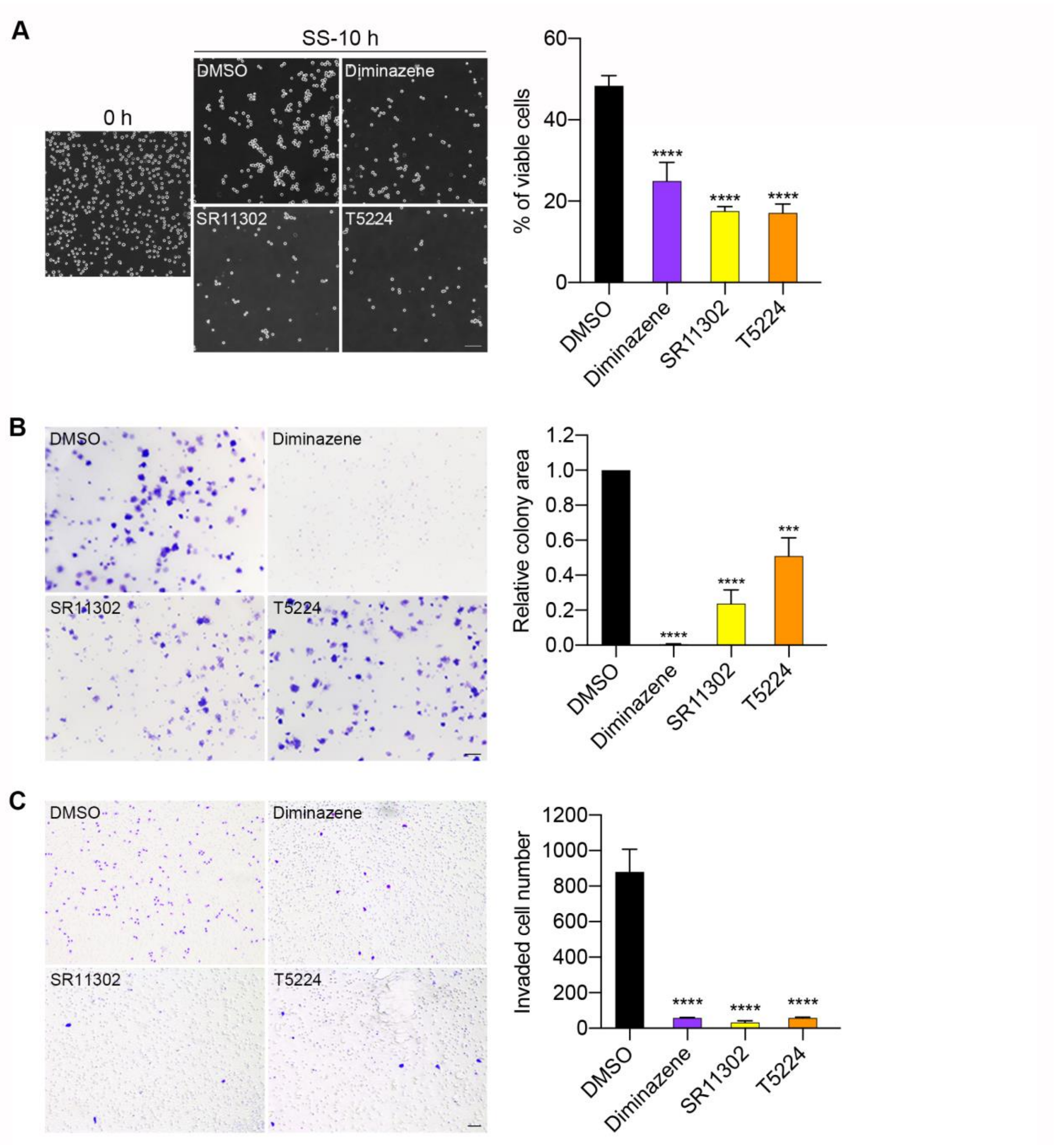

Figure S7. PRSS3 and AP-1 inhibitors significantly suppressed the resistance to SS, cell invasion and colony formation abilities of A-SSP6 cells. (**A**) Representative phase images

and quantified percentage of viable A-SSP6 cells before and after 10 h of circulation. Cells were pretreated with 10 μM PRSS3 inhibitor diminazene or AP-1 inhibitors SR11302 (10 μM) and T5224 (40 μM) for 24 h and then subjected to SS treatment and cocirculated with these inhibitors. Cells were treated with 0.1% DMSO in the control group. Scale bar, 100 μm. (**B**) Representative images and quantified relative colony area of the colonies formed by A-SSP6 cells under treatment with PRSS3 and AP-1 inhibitors. One thousand cells were seeded and allowed to grow for 7 days. The colony area in the control group was considered 1.0, and the relative colony area of each experimental group was calculated. Scale bar, 2 mm. (**C**) Representative images and quantification results of the invaded A-SSP6 cells pretreated with PRSS3 inhibitor and AP-1 inhibitors for 24 h. Twenty thousand cells were seeded in each insert and invaded for 18 h. Indicated inhibitors were also added into the inserts. Scale bar, 100 μm. The quantification results are the means ± SD from three independent experiments. Significant differences were determined by one-way ANOVA. ***$P < 0.001$ and ****$P < 0.0001$.

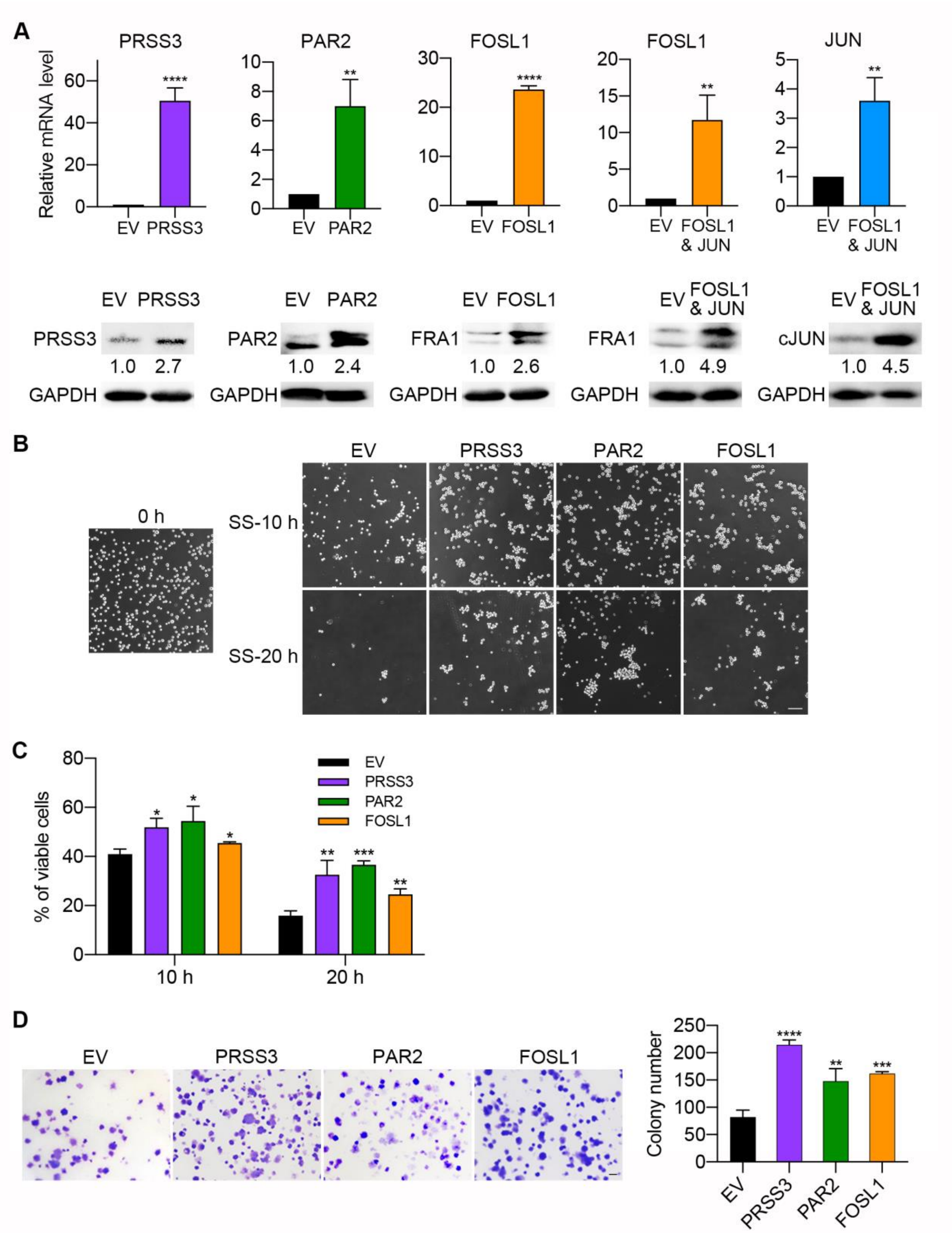

Figure S8. Overexpression of PRSS3, PAR2 and FOSL1 in A549-C3 cells increased the SS survival rate and colony formation ability. (**A**) Efficiencies of overexpressing PRSS3, PAR2, FOSL1 and dual overexpressing FOSL1 and JUN as validated by qPCR (upper) and Western blotting (lower). (**B** and **C**) Representative phase images and quantified percentage of

viable A549-C3 cells overexpressing PRSS3, PAR2 and FOSL1 after 10 h and 20 h of circulation. Scale bar, 100 μm. (**D**) Representative images and quantified number of colonies formed by A549-C3 cells after overexpressing PRSS3, PAR2 and FOSL1. One thousand cells were seeded and allowed to grow for 10 days. Scale bar, 2 mm. The quantification results are the means ± SD from three independent experiments. Significant differences were determined by Student's *t* test (A), two-way ANOVA (C) and one-way ANOVA (D). **P* < 0.05, ***P* < 0.01, ****P* < 0.001 and *****P* < 0.0001.

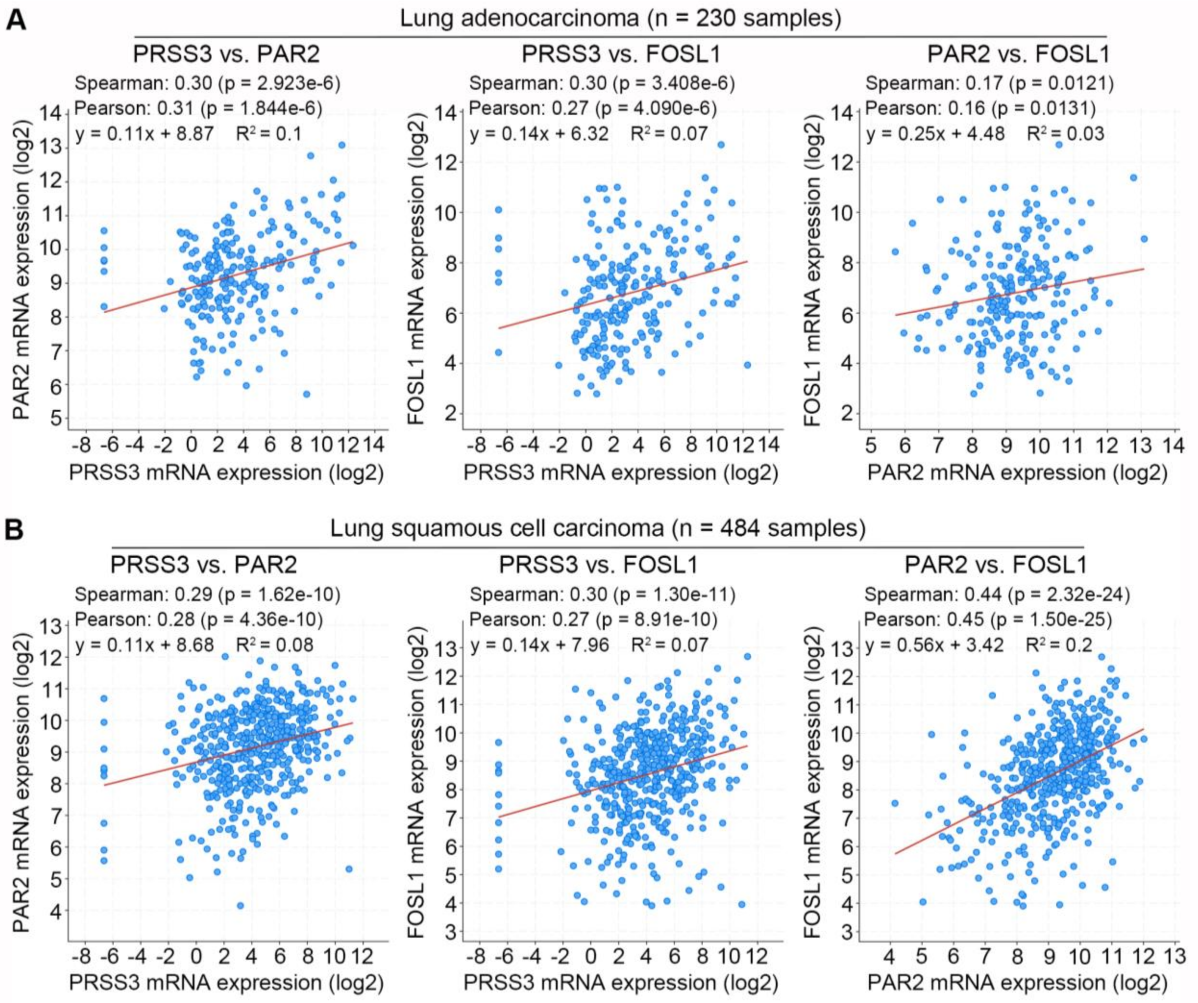

Figure S9. Co-expression of PRSS3, PAR2 and FOSL1 in lung cancer. (**A**) Regression analysis of PRSS3 vs. PAR2, PRSS3 vs. FOSL1 and PAR2 vs. FOSL1 in lung adenocarcinoma performed by cBioPortal. Dataset: Lung adenocarcinoma (TCGA, Nature 2014), *n* = 230 samples. (**B**) Regression analysis of PRSS3 vs. PAR2, PRSS3 vs. FOSL1 and PAR2 vs. FOSL1 in lung squamous cell carcinoma performed by cBioPortal. Dataset: Lung squamous cell carcinoma (TCGA, PanCancer Atlas), *n* = 484 samples.

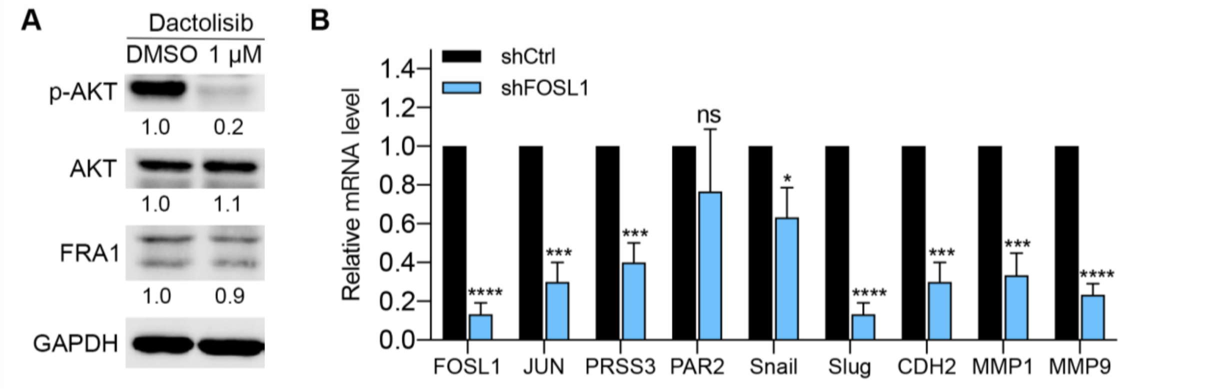

Figure S10. The effects of knocking down FOSL1 on downstream molecules. (**A**) Western blots showing the change in FRA1 after treating A-SSP6 cells with the PI3K inhibitor dactolisib for 24 h. (**B**) Relative mRNA levels of EMT-promoting genes in A-SSP6-shFOSL1 cells compared with A-SSP6-shCtrl cells. The mRNA level of each gene in A-SSP6-shCtrl cells was normalized to 1.0. The quantification results are the means ± SD from three independent experiments. Significant differences were determined by Student's *t* test (B). $^*P < 0.05$, $^{***}P < 0.001$ and $^{****}P < 0.0001$. ns, not significant.

Supplementary tables

Table S1. List of primers for qPCR.

Gene name	Forward (5'-3')	Reverse (5'-3')
GAPDH	CTGGGCTACACTGAGCACC	AAGTGGTCGTTGAGGGCAATG
JUN	TCCAAGTGCCGAAAAAGGAAG	CGAGTTCTGAGCTTTCAAGGT
JUNB	ACGACTCATACACAGCTACGG	GCTCGGTTTCAGGAGTTTGTAGT
JUND	TCATCATCCAGTCCAACGGG	TTCTGCTTGTGTAAATCCTCCAG
FOS	GGGGCAAGGTGGAACAGTTAT	CCGCTTGGAGTGTATCAGTCA
FOSL1	CAGGCGGAGACTGACAAACTG	TCCTTCCGGGATTTTGCAGAT
FOSL2	CAGAAATTCCGGGTAGATATGCC	GGTATGGGTTGGACATGGAGG
PRSS3	CAGCAGCTCACTGCTACAAG	GATCTTGGCCGCATTGATGA
PAR1	CCACCTTAGATCCCCGGTCAT	GTGGGAGGCTGACTACAAACA
PAR2	CTGTGGGTCTTTCTTTTCCGAA	CAAGGGGAACCAGATGACAGA
Snail	TCGGAAGCCTAACTACAGCGA	AGATGAGCATTGGCAGCGAG
Slug	TGTGACAAGGAATATGTGAGCC	TGAGCCCTCAGATTTGACCTG
CDH2	TCAGGCGTCTGTAGAGGCTT	ATGCACATCCTTCGATAAGACTG
MMP1	CTCTGGAGTAATGTCACACCTCT	TGTTGGTCCACCTTTCATCTTC
MMP9	GGGACGCAGACATCGTCATC	TCGTCATCGTCGAAATGGGC

Table S2. List of antibodies.

**Antibody name**	**Company**	**Catalog #**	**Application**
AKT	CST*	4685	WB (1:1000)
p-AKT (Ser473)	CST	4060	WB (1:1000)
cJUN	CST	9165	WB (1:1000), IF (1:100)
p-cJUN (Ser73)	CST	3270	WB (1:1000), IF (1:800)
ERK1/2	CST	4695	WB (1: 2000)
p-ERK1/2 (Thr202/Tyr204)	CST	4370	WB (1: 2000)
FRA1	Abcam	ab124722	WB (1:1000), IHC (1:100)
FRA1	Santa cruz	sc-28310	IF (1:100)
p-FRA1	Affinity	AF4410	WB (1:1000), IF (1:100)
GAPDH	CST	2118	WB (1:1000)
JNK	CST	9252	WB (1:1000)
p-JNK (Thr183/Tyr185)	CST	9251	WB (1:1000)
MMP1	CST	54376	WB (1:1000)
N-cadherin	CST	13116	WB (1:1000)
p38	CST	8690	WB (1:1000)
p-p38	CST	4511	WB (1:1000)
PAR2	Abcam	ab180953	WB (1:1000), IHC (1:100)
PI3K	CST	4249	WB (1:1000)
p-PI3K p85 (Tyr458)/p55 (Tyr199)	CST	4228	WB (1:1000)
PRSS3	abcepta	AP11927c	WB (1:1000), IHC (1:30)
Slug	CST	9585	WB (1:1000)
Snail	CST	3879	WB (1:1000)
Src	CST	2109	WB (1:1000)
p-Src	CST	6943	WB (1:1000)
Vimentin	CST	5741	WB (1:1000)
ZEB1	CST	3396	WB (1:1000)
Goat anti-Rabbit IgG (H+L) Secondary Antibody, Alexa Fluor Plus 488	Invitrogen	A11034	IF (1:100)
Goat anti-Rabbit IgG (H+L) Secondary Antibody, Alexa Fluor Plus 594	Invitrogen	A11037	IF (1:100)
Goat anti-Mouse IgG (H+L) Secondary Antibody, Alexa Fluor Plus 594	Invitrogen	A11032	IF (1:100)
Goat anti-Rabbit IgG (H+L)-HRP Secondary Antibody	Bio-Rad	1706515	WB (1:5000)
Goat anti-Mouse IgG (H+L)-HRP Secondary Antibody	Bio-Rad	1706516	WB (1:5000)

*CST: Cell Signaling Technology

Table S3. List of shRNAs.

shRNA name	TRC Number	Target sequence (5’-3’)
shPRSS3-1		CATCCGCCACCCTAAATACAA
shPRSS3-2		AAAGATTACCAACAGCATGTT
shPAR2	TRCN0000314807	TCTGCTTGTGGTGCATTATTT
shFOSL1-1		GACAAACTGGAAGATGAGAAA
shFOSL1-2		ACACTCATGACCACACCCTCC
shCtrl	SHC002	CAACAAGATGAAGAGCACCAA

Table S4. List of overexpression vectors.

Vector name	Transcript ID	Sequence length
PRSS3	NM_001197097.3	786 bp
PAR2	NM_005242.6	1194 bp
FOSL1	NM_005438.5	816 bp
JUN	NM_002228.4	996 bp
